# Brain-wide perception of the emotional valence of light is regulated by distinct hypothalamic neurons

**DOI:** 10.1038/s41380-022-01567-x

**Published:** 2022-04-28

**Authors:** Mahendra Wagle, Mahdi Zarei, Matthew Lovett-Barron, Kristina Tyler Poston, Jin Xu, Vince Ramey, Katherine S. Pollard, David A. Prober, Jay Schulkin, Karl Deisseroth, Su Guo

**Affiliations:** 1 Department of Bioengineering and Therapeutic Sciences, University of California, San Francisco, CA 94143-2811 USA; 2 Department of Bioengineering, Howard Hughes Medical Institute, Stanford University, Stanford, CA USA; 3 Tianqiao and Chrissy Chen Institute for Neuroscience, Division of Biology and Biological Engineering, California Institute of Technology, Pasadena, CA 91125 USA; 4 Biophysics Graduate Group, University of California, Berkeley, CA USA; 5 Gladstone Institute of Data Science & Biotechnology, San Francisco, CA USA; 6 Department of Epidemiology & Biostatistics, University of California, San Francisco, CA USA; 7 Chan Zuckerberg Biohub, San Francisco, CA USA; 8 Department of Obstetrics & Gynecology, School of Medicine, University of Washington, Seattle, WA USA; 9 Programs in Human Genetics and Biological Sciences, Kavli Institute of Fundamental Neuroscience, The Eli and Edythe Broad Center of Regeneration Medicine and Stem Cell Research, Bakar Aging Research Institute, University of California, San Francisco, CA 94143-2811 USA; 10 Present Address: Neurobiology Section, Division of Biological Sciences, University of California, San Diego, La Jolla, CA USA; 11 Present Address: Invitae Inc., San Francisco, CA USA

**Keywords:** Neuroscience, Genetics

## Abstract

Salient sensory stimuli are perceived by the brain, which guides both the timing and outcome of behaviors in a context-dependent manner. Light is such a stimulus, which is used in treating mood disorders often associated with a dysregulated hypothalamic-pituitary-adrenal stress axis. Relationships between the emotional valence of light and the hypothalamus, and how they interact to exert brain-wide impacts remain unclear. Employing larval zebrafish with analogous hypothalamic systems to mammals, we show in free-swimming animals that hypothalamic corticotropin releasing factor (CRF^Hy^) neurons promote dark avoidance, and such role is not shared by other hypothalamic peptidergic neurons. Single-neuron projection analyses uncover processes extended by individual CRF^Hy^ neurons to multiple targets including sensorimotor and decision-making areas. In vivo calcium imaging uncovers a complex and heterogeneous response of individual CRF^Hy^ neurons to the light or dark stimulus, with a reduced overall sum of CRF neuronal activity in the presence of light. Brain-wide calcium imaging under alternating light/dark stimuli further identifies distinct and distributed photic response neuronal types. CRF^Hy^ neuronal ablation increases an overall representation of light in the brain and broadly enhances the functional connectivity associated with an exploratory brain state. These findings delineate brain-wide photic perception, uncover a previously unknown role of CRF^Hy^ neurons in regulating the perception and emotional valence of light, and suggest that light therapy may alleviate mood disorders through reducing an overall sum of CRF neuronal activity.

## Introduction

Light as a sensory stimulus has biological effects on cognition and mood in addition to its effects on image-forming vision [1]. In humans, light therapy is proven effective for treating mood disorders, linking photic stimuli to emotional regulation [2–4]. Similar effects of light on mood-related behaviors have been observed in animals [5–7], suggesting evolutionarily conserved pathways that remain not well understood.

Neuromodulatory neurons play critical roles in behavioral regulation [8, 9]. How they exert brain-wide impacts remains unclear. The neuropeptide corticotropin releasing factor/hormone (CRF/CRH), first discovered in the early 1980s [10], is an important modulator of stress-associated physiology and behavior [11–15]. Dysregulation of the hypothalamic-pituitary-adrenal (HPA) axis is observed in mood disorders such as major depression [16–20]. CRF-expressing cells are mapped in the nervous system [21, 22]. Hypothalamic CRF is linked to aversive stimuli such as threat or danger [23–25] and their activity is negatively regulated by appetitive stimuli [26]. Despite these advances, the brain-wide effects of CRF neurons remain poorly understood. This is a challenging problem because brain circuits are distributed in distant locations and understanding the entire circuitry requires the ability to monitor neuronal activity throughout the brain.

Larval zebrafish are an attractive model organism for brain-wide circuit level studies [27–37]. As early as five days post fertilization (dpf), they are free-living and need to approach food and avoid predators. Thus, functional circuitry for exploratory reward approaching and anti-predatory avoidance behaviors exists in an accessible and relatively simple vertebrate brain composed of ~100,000 neurons. CRF neurons in larval zebrafish are present in multiple regions analogous to those in the mammalian brain [38–40]. Functionally, they regulate camouflage, a physiological survival reflex [41], and display sensitivity to ethanol [41], hypertonic solutions, and acidic stimuli [36, 42].

A behavior that is simple to characterize in mechanistic detail yet engages complex regulation is the light/dark preference behavior, which reflects the emotional valence of light and is observed across species [7, 43–48]. In mammals, light/dark preference is considered an anxiety-like trait and used to assess the anxiolytic properties of drugs [49]. Larval zebrafish, when presented with a choice for light or dark under well-controlled luminance, tend to spend less time in dark during behavioral tracking on the order of minutes. Intriguingly, treatment of larval zebrafish with anti-anxiety medications reduces dark avoidance [46, 50, 51], whereas stressors enhance the behavior [7, 46, 50, 51]. Dark avoidance also requires intrinsically photosensitive retinal melanopsin neurons (ipRGCs) and habenula function [52].

In this study, we recorded behavioral and brain-wide responses to light/dark stimuli and addressed the role of hypothalamic peptidergic neurons in regulating these responses. We previously gained genetic access to CRF^Hy^ neurons [36]. Here we employ this tool in combination with chemogenetic ablation, optogenetic stimulation, electrophysiology, and in vivo calcium imaging, and demonstrate in free-swimming animals that CRF^Hy^ neurons are necessary and sufficient to drive avoidance of the space where CRF^Hy^ neurons are activated. Inactivation of the *crhb* gene or pharmacological inhibition of CRF receptor-1 activity reduced dark avoidance, indicating direct involvement of CRF neuropeptide. Intriguingly, the role of CRF^Hy^ neurons in promoting dark avoidance is not shared by other hypothalamic peptidergic neurons, even though they are previously shown to play a redundant role in a rapid defensive behavior in head-restrained larval zebrafish [36]. CRF^Hy^ neuronal processes were detected near sensory, motor, and “decision-making’ brain areas, with considerable heterogeneity observed across individual cells. In vivo calcium imaging also uncovered a heterogeneous response of individual CRF^Hy^ neurons to the light or dark stimulus, with a reduced overall sum of CRF neuronal activity in light. In addition to their sensitivity to sensory stimuli, CRF^Hy^ neurons were tuned to motor signals associated with struggle and turn. Finally, brain-wide calcium imaging revealed distinct photic response neuronal types that are distributed throughout the brain. CRF^Hy^ neurons regulated brain-wide photic perception by promoting a neural representation of dark. Brain-wide functional connectivity analysis further broadened our understanding of CRF^Hy^ neurons’ role in balancing brain states that potentially guide action selection between exploration and antipredation in a context-dependent manner.

## Methods

### Experimental model and subject details

All procedures were approved by the University of California San Francisco Institutional Animal Care and Use Committee.

### Zebrafish

The AB-WT strain of zebrafish *Danio rerio* was used in this study. The transgenic lines developed and obtained for this study are detailed Resource Table. Embryos used for imaging experiments were treated with 0.003% of phenylthiourea (PTU) from 22 hpf. Embryos were incubated in E3 embryo medium (5 mM NaCl, 0.17 mM KCl, 0.33 mM CaCl_2_, 0.33 mM MgSO_4_) at 28 °C. The drugs and chemicals were dissolved in the E3 medium as per the concentration mentioned for each experiment.

### Quantification and statistical analysis

Data analysis was performed with custom code written in Python, using NumPy, Scipy, Matplotlib, Python, Seaborn, Statsmodels, Pandas, Scikit-image, Bokeh and Scikit- learn libraries (PMID). MATLAB was used for tracking the tail movement. GraphPad Prism 7 was used preparation of graphs and statistical analysis. All statistical details are described in the Figure captions and result sections, including the exact values of n, what n represents, the statistical tests used and p values for comparisons.

Detailed methods for molecular biology, genetics, CRISPR genome editing, pharmacology, behavior, cortisol measure, optogenetics, calcium imaging, and data analysis can be found in the Supplementary Methods.

## Results

### CRF^Hy^ neuropeptide signaling is selectively required to promote dark avoidance behavior

**Fig. 1:**
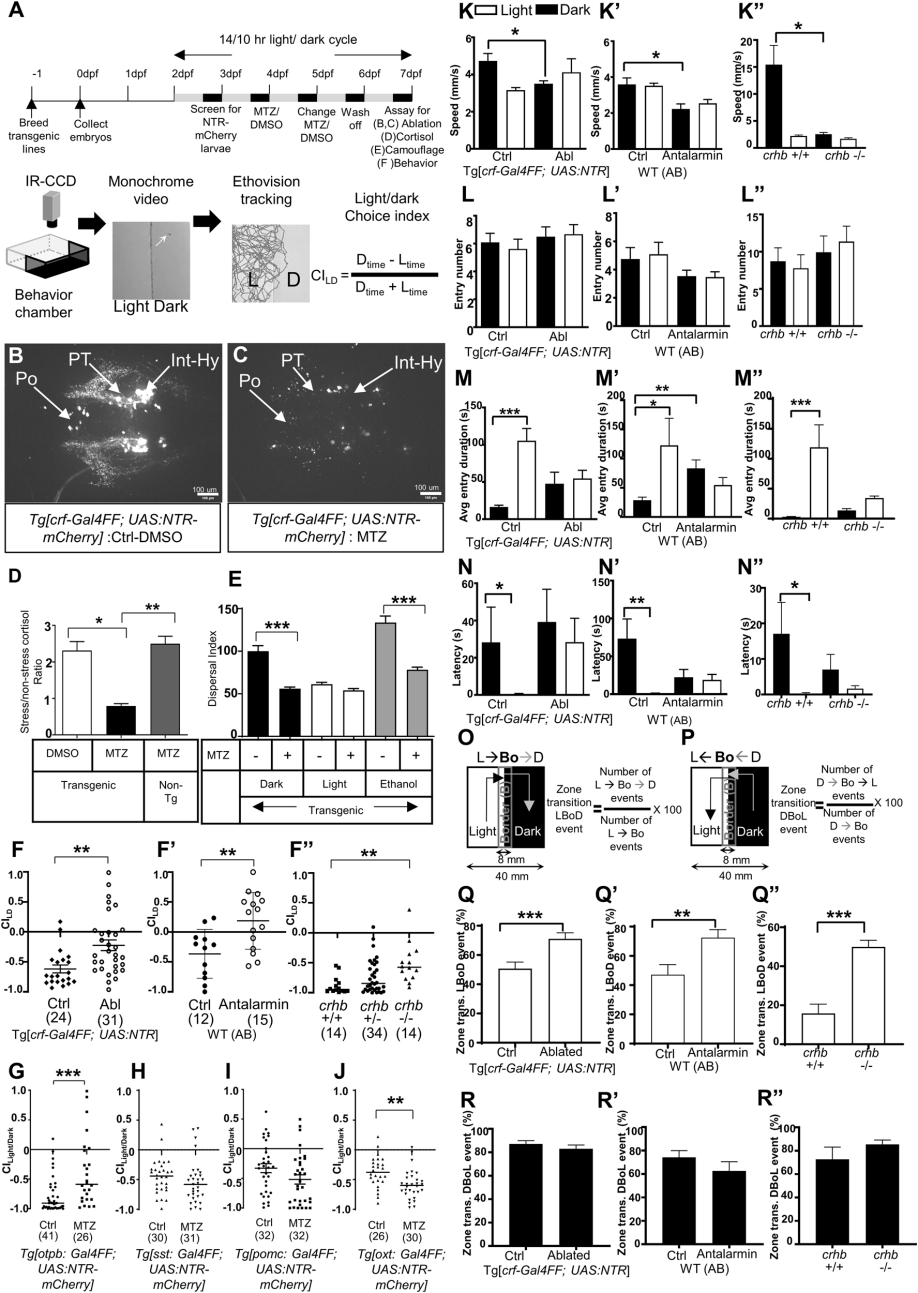
CRF^Hy^ neurons promote dark avoidance by regulating multiple behavioral components. **A** Schematic of the experimental flow. **B**, **C** Images of CRF neurons in transgenic larval zebrafish treated with DMSO (**B**) and Metronidazole (MTZ) (**C**). **D** Ratio of cortisol level (stress/baseline) in control and CRF^Hy^-ablated subjects, Kruskal–Wallis test, Dunn’s multiple comparisons test, *n* = 6 per group, *p < 0.05, **p < 0.01. **E** Camouflage respons**e** in control and CRF^Hy^ -ablated subjects upon light, dark or ethanol treatment. Ordinary one-way ANOVA, Sidak’s multiple comparison test, *n* = 16 per group, ***p < 0.001. Error bars represent SEM. **F**–**F”** Light–dark choice index (CI_LD_) upon CRF^Hy^ neuronal ablation (**F**), CRF receptor 1 antagonist treatment (**F’**), and in *crhb*^*−/−*^ mutants (**F”**), in comparison to corresponding sibling controls. Note that the behavior is sensitive to genetic backgrounds. **G**–**J** CI_LD_ of larvae with ablation of *otpb*-expressing neurons (**G**), somatostatin neurons (**H**), *pomc* pituitary neurons (**I**), and oxytocin neurons (**J**), with corresponding sibling control (non-ablated) larvae. For all graphs, normality test was performed to decide gaussian distribution and accordingly Mann–Whitney test for (**F**, **F’**, **G**, **H**, **I**), and unpaired *t* test with Welch’s correction for (**J**) and ANOVA followed by comparison test was performed (Kruskal–Wallis test and Dunn’s multiple comparisons test) for (**F”**), was performed, *p < 0.05, **p < 0.01, ***p < 0.001, numbers with parathesis indicate sample size, error bars representing SEM. **K**–**N** Kinematics of behavior upon CRF^Hy^ ablation, CRF receptor antagonist treatment, or in *crhb*^*−/−*^ mutants in comparison to corresponding control siblings; data are shown in light (white bars) and dark zones (black bars). Comparison of swim speed (**K**–**K”**), numbers of zone entries (**L**–**L”**), time spent in zone upon entry (**M**–**M”**), Latency to first zone entry (**N**–**N”**). **O**, **P** Schematic showing transition at the boundary of light–dark zone for events of crossover to dark zone (Light- > Border- > Dark, **O**) and crossover to light zone (Dark- > Border- > Light: **P**) with formula to calculate % zone transition LBoD and DBoL events respectively. **Q**–**Q”** Bar graphs showing comparison of percent zone transition events from Light -> Bo- > Dark (LBoD) upon CRF^Hy^ ablation (**Q**), CRF receptor antagonist treatment (**Q’**) and in *crhb*^*−/−*^ mutants (**Q”**) with respective controls. **R**–**R”** Bar graphs showing comparison of percent zone transition events from Dark- > Bo- > Light (DBoL) upon CRF^Hy^ ablation (**R**), CRF receptor antagonist treatment (**R’**) and in *crhb*^*−/−*^ mutants (**R”**) with respective controls. Sample size is displayed in Panel (**F**–**F”**). For all graphs, a normality test was performed to decide gaussian distribution and accordingly Mann–Whitney test (for **Q**, **R**, **R”**), Unpaired *t* test with Welch’s correction (for **Q’**, **Q”** and **R’**) and ANOVA followed by comparison test was performed (Kruskal–Wallis test and Dunn’s multiple comparisons test for **K**–**K”**, **L**–**L”**, **M**–**M”**, **N**–**N”**), *p < 0.05, **p < 0.01, ***p < 0.001, error bars representing SEM.

Under well-controlled luminance history and surrounding, larval zebrafish of 5–7 dpf display a tendency to avoid dark in a light/dark preference behavioral paradigm (Fig. 1A; Video S1): Individual animals were monitored for ~8 min during free exploration of a half-light/half-dark arena. This behavior is sensitive to anxiolytics and environmental stressors [7, 51]. The extent of dark avoidance likely results from two competing drives: one is a natural tendency to explore, and the other is to avoid a potential threat (e.g., the dark side, which may signify coldness or predator shadow). The resulting outcome can be quantified using a light/dark choice index (CI_LD_): −1 denotes 100% time in light, 0 denotes no preference, and 1 indicates 100% time in dark. To investigate a possible role of CRF^Hy^ neurons in dark avoidance behavior, we gained genetic access to these neurons using a cross-species enhancer detection approach (Fig. S1) [36] and employed the nitroreductase/metronidazole (NTR/MTZ) system [53, 54] for chemogenetic ablation. The genetically accessible CRF^Hy^ neurons are mostly located in the intermediate hypothalamus (Int-Hy), with minor populations detected in the preoptic nucleus (Po) and posterior tuberculum (PT); upon addition of MTZ, all three groups were effectively ablated (Fig. 1B, C). Consistent with a known role of CRF in regulating the hypothalamic-pituitary physiological stress axis, CRF^Hy^ -ablated animals showed a blunted cortisol level upon stress (Fig. 1D) and decreased camouflage response in dark or upon ethanol stimulation (Fig. 1E). These observations validate the efficacy of ablation and confirm the requirement of CRF^Hy^ neurons in physiological stress response.

We further observed that at the behavioral level, CRF^Hy^-ablated animals displayed significantly increased population mean CI_LD_ compared to sibling controls (Fig. 1F), indicating a decreased dark avoidance. CRF^Hy^ neurons, together with other peptidergic neurons in the hypothalamus, co-release the excitatory neurotransmitter glutamate that mediates a fast-timescale (within seconds) defensive behavioral response [36]. To determine whether the role of CRF^Hy^ neurons in dark avoidance behavior is mediated by the neuropeptide CRF, we carried out pharmacological inhibition of the CRF receptor 1 (CRF-R1) with the antagonist Antalarmin; we also performed genetic disruption of the *crhb* gene that encodes the CRF neuropeptide (Fig. S2). In both cases, dark avoidance behavior was significantly attenuated compared to respective sibling controls (Fig. 1F’-F”), indicating that, different from the fast-timescale defensive response, the choice to avoid dark in an 8-minute timescale requires CRF neuropeptide signaling.

To address whether other hypothalamic peptidergic neurons play a role in dark avoidance, we chemogenetically ablated oxytocin (OXT) neurons using *Tg[oxt:Gal4]* [55] or somatostatin (SST) neurons using *Tg[sst3:Gal4]* [56]. A line with broad expression in hypothalamic Otpba neuroendocrine neurons including CRF^Hy^ neurons (*Tg[otpba:Gal4])* [57, 58] was also used. We also tested whether the role of CRF in dark avoidance is mediated through its effect on proopiomelanocortin (POMC) neurons in the pituitary gland by ablating these neurons using *Tg[pomc:Gal4FF]*. Ablation of Otpba-expressing neurons had the same effect as ablating CRF^Hy^ neurons (Fig. 1G), while ablation of SST or POMC neurons had no effect (Fig. 1H, I). Intriguingly, ablation of OXT neurons significantly increased dark avoidance (Fig. 1J). Taken together, hypothalamic neuropeptidergic neurons play distinct roles in regulating dark avoidance.

While simple to quantify, dark avoidance is a complex behavior that results from a balance between exploration and anti-predation and involves moment-to-moment decision-making. To further understand the role of CRF^Hy^ in this complex behavior, we carried out kinematic analyses of several behavioral components, including zone transition probability at the border as a way of measuring “decision-making” (Fig. 1K–R). Disruption of CRF (due to ablation of CRF^Hy^ neurons, pharmacological inhibition of CRF-R1, or genetic mutation of the *crhb* gene) resulted in a decrease of speed in the dark zone (Fig. 1K–K”) but the zone entry number was unaffected (Fig. 1L–L”). While respective controls have a longer average entry duration in the light than the dark zone, CRF-disrupted groups did not (Fig. 1M–M”). Respective controls also showed a significantly shorter latency to enter the light than the dark zone, but CRF-disrupted groups did not (Fig. 1N–N”). Lastly, we analyzed how CRF might affect a decision at the border. The border zone is defined as an 8 mm region that spans the light/dark boundary. The total border zone width is 1/5 that of the arena (40 mm) and twice the length of larval zebrafish (~4 mm). Transitions from both light->dark and dark->light were examined (Fig. 1O, P). When entering the border zone from the light side, CRF-disrupted animals showed an increased probability of entering the dark zone (Fig. 1Q–Q”). However, when entering the border zone from the dark side, no significant differences were observed between CRF-disrupted and control groups (Fig. 1R–R”). These findings reveal a requirement of CRF^Hy^ in promoting dark avoidance, by acting on multiple behavioral components, including decreasing the duration of dark entry, reducing the latency to enter the light side, and biasing a decision toward avoiding dark.

### Optogenetic activation of CRF^Hy^ neurons is sufficient to drive avoidance behavior

**Fig. 2:**
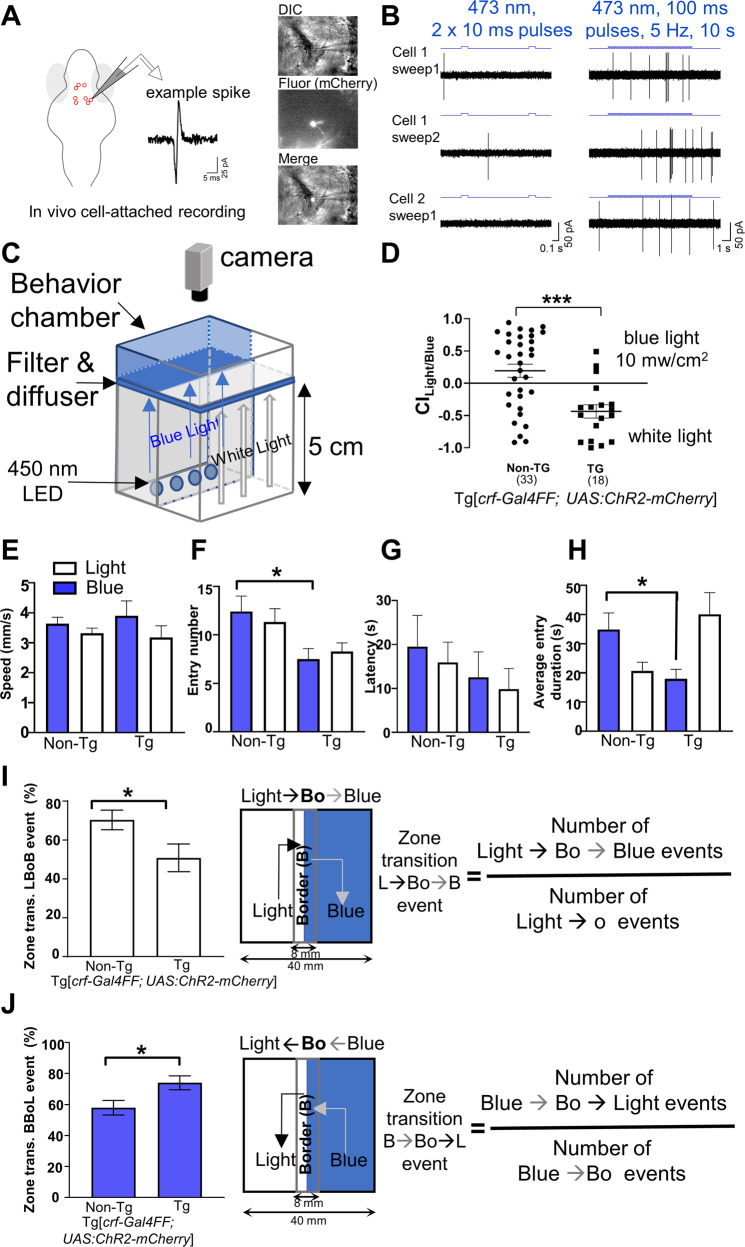
Optogenetic activation of CRF^Hy^ neurons promotes avoidance behavior. **A** Schematic of in vivo cell-attached recording of CRF neurons expressing ChR2-mcherry. **B** Recordings from CRF neurons upon stimulation with 473 nm light at 3 mW shows that single pulses do not drive spiking reliably, but prolonged stimulation causes robust spiking. **C** Schematic of the optogenetic behavior setup. **D** White vs blue light choice index for transgenic and non-transgenic sibs. **E**–**H** Kinematics of choice behavior for white light (white bars) and blue light zones (blue bars), for swim speed (**E**), entry numbers (**F**), latency (**G**), and entry duration (**H**). **I**, **J** Zone transition decision at the boundary of white light-blue light zone: bar graphs showing precent of zone transition events and schematic showing transition along with formula to calculate % zone transition events for cross over to blue zone (Light- > Bo- > Blue, **I**) and to light zone (Blue- > Bo- > Light, **J**). For all graphs, normality test was performed to assess gaussian distribution and Mann–Whitney test (for **D**, **I**, **J**), Kruskal–Wallis test and Dunn’s multiple comparisons test (for **E**–**H**) were performed. *n* = 35 (non-TG), 36 (TG),*p < 0.05, ***p < 0.0001, error bars representing SEM.

Next, we determined whether activation of CRF^Hy^ neurons was sufficient to drive avoidance, using the transgenic line *Tg[crf:Gal4FF; UAS:ChR2-mCherry]*. We first verified by electrophysiology that CRF^Hy^ neurons were activated by 473 nm blue light delivered for 10 s (100 ms pulses, 5 Hz) (Fig. 2A, B). We then constructed a behavioral chamber where half of the chamber was illuminated with 450 nm blue light and the other half illuminated with white light (Fig. 2C). We tuned the blue light intensity by adjusting the distance between the light source and the behavioral chamber. At 10 mW/cm [2], non-transgenic siblings displayed a slight preference for the blue-lit side but *Tg[crf:Gal4FF; UAS:ChR2-mCherry]* animals significantly avoided it (Fig. 2D). Further kinematic analysis uncovered that the transgenic animals made fewer entries into the blue-lit side (Fig. 2F) and spent less time in it (Fig. 2H). Swim velocity and latency to enter zones were not significantly different between control and transgenic animals (Fig. 2E, G). Analysis of “decision-making” at the border zone uncovered a significantly reduced probability of transitioning from the white to the blue light (Fig. 2I) and increased probability of transitioning from blue to the white light in the transgenic animals (Fig. 2J). Together, optogenetic activation of CRF^Hy^ neurons is sufficient to promote avoidance of the space where CRF^Hy^ neurons are activated.

### Single CRF^Hy^ neuron projection analysis reveals broad yet heterogeneous connections near sensory, motor, and decision-making brain areas

**Fig. 3:**
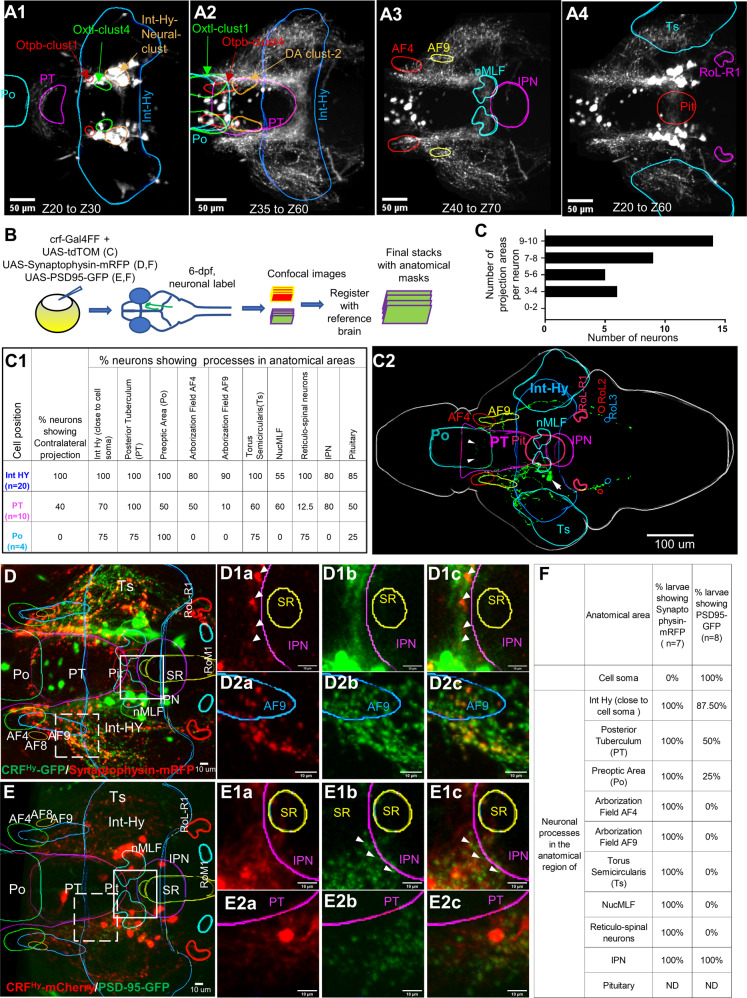
Single neuron projection analyses reveal broad connections of individual CRF^Hy^ neurons. **A1**–**A4** Images of selected z-plane stacks averaged from 10 larval brains (6–7 dpf) registered with the z-brain atlas and overlaid with anatomical masks. CRF neurons are near Otpb-cluster 1 and Oxt-cluster 4 and occupies a position in the Intermediate Hy neural cluster (A1). CRF neurons in preoptic and posterior tuberculum are near oxtl-cluster 1, Otpb-cluster 4 and Dopaminergic neuron cluster 2 (**A2**). CRF neuronal processes reach arborization fields AF4, AF9, nucleus of medial longitudinal fascicle (nMLF) and inter-peduncular nucleus (IPN) (A3). CRF neuronal processes are detected in the Torus semicicularis (Ts), pituitary (Pit), and reticular spinal motor neurons in Rol1-R1 (A4). **B** Schematic of single neuron labeling and registration. Similar scheme was followed with injection of UAS:PSD95-GFP or UAS:Synaptophysin-RFP into *Tg[CRF*^*Hy*^*:GAL4FF]* embryos. **C**–**C2** Single neuron labeling and projection analysis. A histogram showing the number of CRF neurons (x-axis) with the number of projection areas (*y*-axis) analyzed by single neuron labeling (**C**). Table showing number of CRF neurons imaged in intermediate hypothalamus (Int-Hy), Posterior Tuberculum (PT) and Pre-optic (Po) with their neuronal processes identified in anatomical areas (**C1**). An example single neuron-labeled stack registered with Z-brain and maximum intensity projection overlaid with anatomical masks. The cell soma is positioned in Int-Hy (pointed by a white arrow) and its processes are detected both ipsi- and contra-laterally, with the crossover to the contralateral side pointed by arrowheads (C2). **D** Presynaptic labeling of transgenic CRF^Hy^ neuronal processes. Maximum intensity projection of z-plane images from CRF^Hy^ transgenic larva expressing synaptophysin-mRFP (red) and cytoplasmic GFP, overlaid with Z-brain atlas anatomical mask outlines after registration. D1a–D1c zoomed-in images of solid line square box in (**D**) created with maximum intensity projection of selected z-plane images, showing neuronal process expressing synaptophysin-mRFP (D1a), GFP (D1b) and overlap (D1c) near IPN; a few synaptophysin-mRFP puncta detected in the region in all subjects. D2a–D2c zoomed-in images of dashed line square box in (**D**) created with maximum intensity projection of selected z-plane images, showing neuronal process expressing synaptophysin-RFP (D2a), GFP (D2b) and overlap (D2c); synaptophysin puncta was detected in neuronal processes near AF9. **E** Post-synaptic labeling of transgenic CRF^Hy^ neuronal process. Maximum intensity projection of z-plane images from CRF^Hy^ transgenic larva expressing PSD95-GFP (green) and cytoplasmic mCherry, overlaid with the anatomical masks after registration. E1a–E1c zoomed-in images of solid line square box in (**E**) created with maximum intensity projection of selected z-plane images, showing neuronal processes expressing mCherry (E1a), PSD95-GFP (E1b) and overlap (E1c) near IPN. E2a–E2c Zoomed-in images of dashed line square box in (**E**) created with maximum intensity projection of selected z-plane images, showing neuronal processes expressing mCherry (E2a), PSD95-GFP(E2b) and overlap (E2c); PSD95-GFP puncta was detected in neuronal processes in PT**. F** Table showing precent larvae with detection of synaptophysin-mRFP and PSD-95-GFP near cell soma and neuronal processes in various projection areas. Abbreviations: Refer to Table S3.

How do CRF^Hy^ neurons promote dark avoidance? To begin to address this question, we mapped the anatomical location of their cell bodies and neuronal processes. By registering images of CRF^Hy^ transgenic animals to the Z-brain atlas [59] (Fig. S3), we found that most of the labeled CRF^Hy^ neurons were in the intermediate hypothalamus (Int-Hy) near the diencephalic Otp Cluster 1 and the oxytocin-cluster 4 (Fig. 3A1). This anatomical location suggests that Int-Hy CRF^Hy^ neurons may correspond to the magnocellular population of CRF^Hy^ neurons in the mammalian brain, which are also close to oxytocinergic cells [60]. Smaller numbers of labeled CRF^Hy^ neurons were present in the preoptic (Po) and posterior tuberculum (PT) (Fig. 3A2–4, also see Fig. 1B).

CRF^Hy^ neuronal processes were detected in the visual [e.g., Arborization Fields (AF) AF4, AF9], auditory [e.g., Torus Semicirularis (Ts)] (Fig. 3A2, A3), pre-motor (e.g., nMLF) and motor areas (e.g., reticulospinal motor neurons), as well as the pituitary (Fig. 3A4). The observations of CRF processes near visual and motor areas were verified by DiI injection into the retina and dextran backfilling from the spinal cord (Fig. S4A–E). The presence of CRF processes near the pituitary was verified by adrenocorticotropic hormone (ACTH) antibody labeling (Fig. S4F). CRF neuronal processes were also detected in the interpeduncular nucleus (IPN) (Fig. 3A3), which receives direct inputs from the habenula (Hb), an important decision-making brain area [61].

The finding that CRF^Hy^ neurons extend processes to sensory, motor, and decision-making areas are in line with the notion that neuromodulators such as CRF can act at different sites within a circuit to coordinate functional outputs [8]. Does each CRF^Hy^ neuron connect to multiple target areas, or alternatively, is such broad connectivity a collective feature of the CRF^Hy^ neuronal group? To differentiate these possibilities, we performed single neuron projection analysis using sparse labeling in transgenic larvae followed by whole brain registration and reconstruction (Fig. 3B, and Video S2). We found that most CRF^Hy^ neurons projected to multiple target areas (Fig. 3C). For instance, the processes of a single Int-Hy CRF neuron were detected in ten different brain regions (Fig. 3C1, C2). When comparing the three subgroups of CRF^Hy^ neurons (i.e., Po, PT, and Int-Hy), we found that processes of each of these sub-groups were detected in the hypothalamus, the auditory area Ts, reticulospinal neurons, and pituitary. Intriguingly, neuronal processes from Int-Hy and PT but not Po were detected in the visual fields, the pre-motor nMLF, and IPN. Taken together, our data indicate that most CRF^Hy^ neurons project to a broad set of brain regions, albeit heterogeneity exists across individual neurons.

Finally, to determine the pre- vs. post-synaptic identity of CRF^Hy^ neuronal processes, we performed co-labeling with synaptophysin-mRFP (to mark presynaptic terminals) (Fig. 3D) and PSD95-GFP (to mark post-synaptic terminals) (Fig. 3E). Presynaptic terminal co-labeling was detected in most CRF^Hy^ neuronal processes near visual, motor, and “decision-making” areas (Fig. 3D1–D2, selected 10-Z). Post-synaptic terminal co-labeling was detected in CRF^Hy^ neuronal processes within the hypothalamus, on CRF^Hy^ neuronal soma, and intriguingly also near the IPN (Fig. 3E1–E2, selected 10-Z), suggesting possible reciprocal connections between CRF^Hy^ and IPN. Together, these analyses uncover at single-cell resolution that CRF^Hy^ neurons extend processes near sensory (e.g., both visual and auditory), motor (e.g., pre-motor nMLF and reticulospinal MNs), and decision-making (e.g., IPN) brain areas.

### In vivo calcium imaging uncovers a heterogeneous response of individual CRF^Hy^ neurons to light/dark stimuli, with a reduced overall sum of CRF neuronal activity in light

**Fig. 4:**
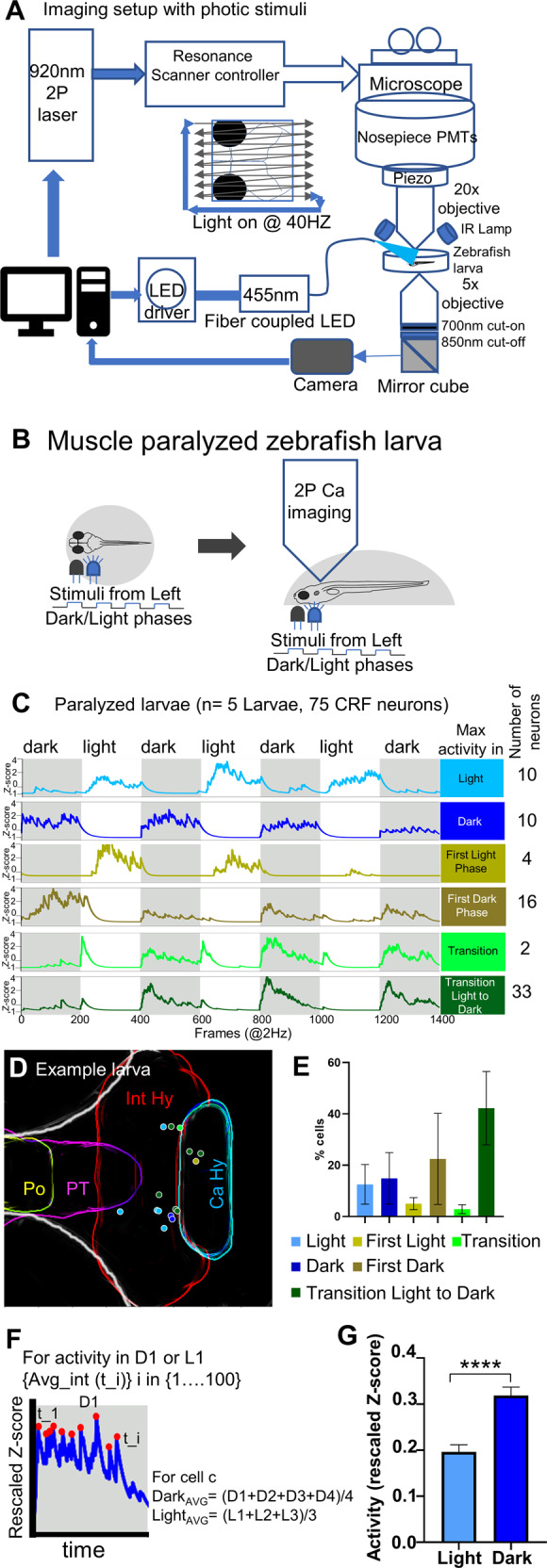
In vivo calcium imaging of CRF^Hy^ neuronal activity upon light/dark stimuli. **A** Schematic of the setup that enables simultaneous calcium imaging with two-photon resonance scanning microscope and light stimulus delivery. Light stimuli were pulse delivered during the y-flyback of galvo scanner such that the light does not interfere with GCaMP imaging. **B** Schematic showing neuronal activity imaging in paralyzed and completely embedded larva. The blue LED (455 nm) light was pulsed as visual stimuli in alternate light and dark phases, each lasting 100 seconds. **C** CRF^Hy^ neuronal activity imaged in paralyzed larvae, classified into six classes in correlation with phases of photic stimuli. side bars show activity classes, and corresponding Z-score plots show CRF^Hy^ activity for each class, gray and white blocks representing dark and light phases respectively. The number of CRF^Hy^ neurons in each photic response class was shown on the right. **D** Schematic showing the position of CRF neurons as colored circles corresponding to each photic response class in an example larva. **E** A plot showing the percentage distribution CRF^Hy^ neurons among the six photic response classes (*n* = 5 larvae). **F** Schematic showing the calculation of activity response in each dark/light phase; (**G**) the plot showing the average activity of CRF^Hy^ neurons in light vs dark, Mann–Whitney test, ****p < 0.0001, *n* = 75 neurons from 5 larvae, error bars representing SEM.

For CRF^Hy^ neurons to promote dark avoidance, they must receive photic information, either directly or indirectly. To determine whether light/dark stimuli impact CRF^Hy^ neuronal activity, we performed in vivo calcium imaging in head-restrained and muscle-paralyzed *Tg[crf:Gal4FF; UAS:GCaMP6s]* animals. Alternating periods of dark or light (100 seconds each) were presented during 2-photon imaging: To avoid tripping the photomultiplier tube (PMT), a pulsed light at 40 Hz was delivered (Fig. 4A, B). Possibly due to the relative weaker fluorescence of GCaMP6s than GFP, only CRF^Hy^ neurons in the Int-Hy were visible and therefore recorded in the transgenic line. Six distinct light/dark response CRF types were detected (using a custom method as described in next section), suggesting heterogeneity in their activity profiles. Most neurons displayed high activity in dark (including all dark phases, first dark phase, and transition from light to dark, *n* = 59/75 neurons, from FIVE animals) (Fig. 4C–E). By calculating the sum of neuronal activity in dark vs. light, we uncovered a significantly higher activity of CRF neurons in dark than in light (Fig. 4F, G). These results indicate that light reduces the overall sum of CRF^Hy^ neuronal activity.

To simultaneously track CRF^Hy^ neuronal activity and behavioral output upon light/dark stimuli, we performed in vivo calcium imaging in head-restrained tail-free larval zebrafish (Fig. S5A). To characterize motor patterns, we first analyzed tail movement tracking videos using a previously described Matlab script [62]. The tail tip angles calculated from this script were further analyzed using custom python scripts to classify tail movement events into three main categories (Fig. S5B–E): Struggle is characterized as vigorous bi-directional tail swing (Fig. S5C–C’), swimming is defined as small-angled rhythmic bi-directional tail movement (Fig. S5D–D’), and turn is a strong unidirectional tail swing (Fig. S5E–E’). We found that under the head-restrained tail-free setting, CRF^Hy^ neuronal activity was strongly tuned to tail movements (Fig. S5F). The occurrences of struggles, swim, and turns were not significantly different in dark vs. light (Fig. S5G).

Intriguingly, by examining the correlations between peak CRF^Hy^ neuronal activity surrounding the tail movement events, we found that individual CRF neurons could be classified as being mostly active either before, concurrent, or after the tail movement events, with most CRF neurons showing peak activity after the tail movement events (See Fig. S5G–G”). These observations further highlight the functional heterogeneity of CRF^Hy^ neurons.

### Brain-wide calcium imaging uncovers distributed coding of photic stimuli and vigorous motion

**Fig. 5:**
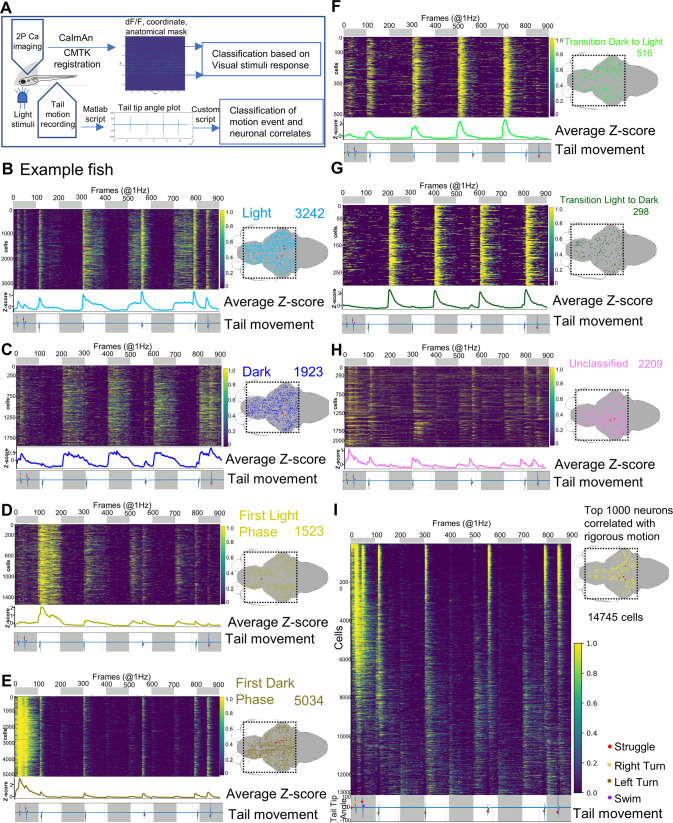
Brain-wide calcium imaging uncovers distributed coding of photic stimuli and motor information. **A** Schematic showing the setup and pipeline of brain-wide neural activity data processing. Transgenic larva *Tg[HuC:H2B-GcaMP6s;crf:Gal4FF;UAS:GCaMP6s;UAS:NTR-mCherry]* is embedded in low melting agarose leaving the tail free. GCaMP imaging, tail movement recording, and light stimulus delivery is similar to the setup described in Fig. 4A. The calcium imaging data are processed through CaImAn and ROI (neuronal nuclei) coordinates from CaImAn analysis were registered to the Z-brain atlas template through CMTK registration. **B**–**H** Example of brain-wide activity heatmaps showing seven photic response neuronal types (left) and their distributions in the brain (right). Dotted squares in the 2D brain schematics represent the field of view of imaging; red dots show the position of CRF^Hy^ neurons. The number of cells scored in each photic response class is denoted in the top right corner. The top bar shows dark (grey) and light (white) phases. At the bottom of each heatmap plots is average z-score activity plots for corresponding classes along with the tail movement plot showing tail tip angles (*y*-axis). **I** Example of brain-wide activity heatmaps arranged in order of correlation with tail moments. The bottom shows the tail movement plot with light and dark phases indicated. On the right is a 2D brain schematic, showing corresponding brain-wide distributions of top 1,000 neurons showing vigorous motion-correlated neuronal activity; red dots show the position of CRF^Hy^ neurons.

So far, we have shown that CRF^Hy^ neurons promote dark avoidance in free swimming larval zebrafish; they extend processes to sensory, motor, and decision-making brain areas, and are more active in dark than in light. To further understand how photic stimuli and vigorous motion are encoded in the brain, we performed brain-wide calcium imaging upon delivery of light/dark stimuli to head-restrained tail-free larval zebrafish (Fig. 5A). Recording from restrained and behaving animals is a powerful approach to simultaneously track neural activity and behavior and has been used across species. However, it is worth noting that restraining is a strong threat and can impact brain state and behavior. The vigorous motion (i.e., struggle and turn) detected in head-restrained tail-free animals is likely an escape response to head restraining.

Transgenic *Tg[HuC-H2B-GCaMP6s, crf-Gal4FF, uas-GCaMP6s, uas-NTRmCherry]* animals were used. Since the HuC promoter-driven H2B-GC*a*MP6s was undetectable in most int-Hy CRF^Hy^ neurons, the transgenes *crf-Gal4FF, uas-NTRmCherry, and uas-GCaMP6s* were used to detect the activity of CRF^Hy^ neurons during brain-wide calcium imaging. Areas including the forebrain, midbrain, and anterior hindbrain were imaged. We established a data processing pipeline that obtained single-cell resolution neuronal activity data of over 14,000 neurons per subject (14,972 ± 357, *n* = 10 subjects) and detected neuronal correlates to both photic stimuli and tail movements (Fig. S6A) (Video S3). We also used the image registration pipeline (Fig. S3) that enabled us to assign each neuron to an anatomical mask in the Z-brain atlas and compare neuronal activity profiles of each anatomical area across different individuals. We found that CRF^Hy^ neurons, as described earlier, showed distinct photic responses, and were also activated during vigorous motion (marked by red dots in Fig. 5B–I, right). These findings further validated our brain-wide analytical approach.

We next aimed to classify neurons brain-wide based on their tuning properties to photic stimuli. While it is possible to use regression analysis, one limitation of such method is its bias toward cells with sustained stimulus responses; cells transiently active at the onset of the stimulus will be missed. Here we employed a different algorithm known as the heap que algorithm [63], which not only enabled the identification of transiently active cell types but also reduced computation complexity. We computed the nlargest values (Fig. S6B–D) to detect neuronal activity associated with light/dark phases. By classifying each neuron based on their top 10% z-scored activity values in each light or dark phase (Fig. 5B–H), we uncovered seven different photic response classes: (1) activated in light (Fig. 5B, 1940 ± 171); (2) activated in dark (Fig. 5C, 2553 ± 232); (3) activated in first light phase (Fig. 5D, 1683 ± 177); (4) activated in first dark phase (Fig. 5E, 5787 ± 428); (5) activated during transition from dark to light (Fig. 5F, 190 ± 40); (6) activated during transition from light to dark (Fig. 5G, 174 ± 21); (7) unclassified (Fig. 5H, 2643 ± 197), which contains neurons not responsive to light or dark, or having mixed properties [64]. Neurons activated during light/dark transitions were notably fewer than other classes. All classes of photic responsive neurons are present throughout the brain (Fig. 5B–H, right), suggesting distributed coding of photic information.

It is worth noting that light-tuned (Fig. 5B) and first dark phase-tuned (Fig. 5E) neuronal types were also strongly activated during vigorous motion, whereas other photic response types are weakly activated during vigorous motion (Fig. 5C, D, F, G). Using the same algorithm for classifying photic response types, we classified neurons based on their correlation with vigorous tail movements and observed that most neurons in the brain were activated during vigorous motion. Among them, the top 1,000 neurons were shown distributed across brain regions (Fig. 5I). Thus, both photic and motor variables are encoded in a distributed fashion in the brain; specific neuronal response types are identified that are activated in light, dark, first light, first dark, or transitions between light/dark.

### CRF^Hy^ neurons suppress light representation in selective and distributed brain areas

**Fig. 6:**
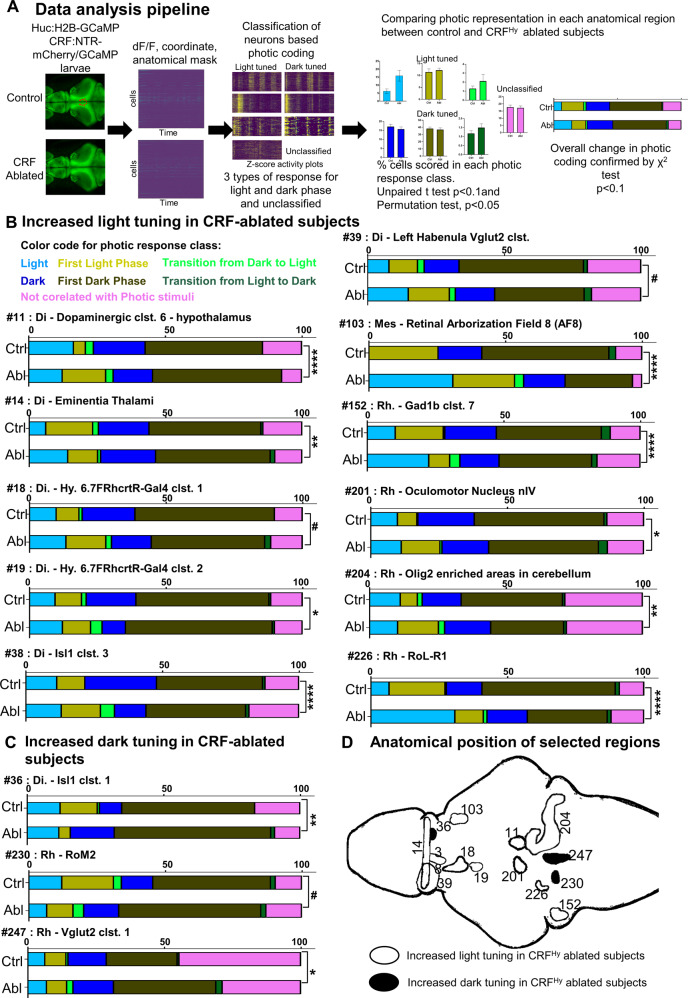
CRF^Hy^ neurons decrease the representation of light in a highly selective, distributed set of brain areas. **A** Schematic showing the data processing pipeline. Control and CRF^Hy^-ablated *Tg[HuC:H2B-GcaMP6s, crf:Gal4FF;UAS:GCaMP6s,UAS:NTR-mCherry]* larvae were subjected to brain-wide calcium imaging as described in Fig. 5A. The data processing yields the information of each neuron’s activity (dff), coordinates and anatomical regions (mask) it belongs to. Neurons were classified based on their max activity with respect to light and dark phases. For each anatomical region, the percent of cells in each photic class were scored and compared between control and CRF^Hy^-ablated subjects. Anatomical regions showing significant difference in at least one of the photic classes were selected. The comparison of overall distribution of cells in all photic classes in the significant anatomical regions was further carried out with χ^2^ test. **B** Color codes are used to represent each photic class. Bar graphs showing comparison of proportion of cells belonging to each photic response class in control vs CRF^Hy^-ablated subjects. Increased tuning to the light was observed in CRF^Hy^-ablated subjects for 11 brain areas, with ID number 14, 39, 103, 226 having a significant increase of “light” response type, ID number 11, 18, 201, 204 having significant increase of “First light” response type, ID number 152 having a shift from First light to light response type, whereas ID number 19 and 38 having a significant decrease in “dark” response type. **C** Bar graphs for anatomical regions showing increased tuning to dark in the CRF^Hy^-ablated subjects. Anatomical region with ID 36 showed a significant increase in “dark” response type whereas anatomical region with ID 230 and 247 showed a significant increase in “First dark” response type. For (**B**) and (**C**) χ^2^ test, #*p* < 0.1, *p < 0.05, **p < 0.01, ***p < 0.001, ****p < 0.0001, *n* = 10 larvae per group. **D** Schematic showing the position of anatomical regions presented in (**B**) and (**C**). The anatomical mask outlines are shown unilaterally (except for #14 and #39).

Given that CRF^Hy^ neurons promote dark avoidance in free-swimming animals, we asked whether they might do so in part by altering photic perception. Brain-wide calcium imaging was carried out in head-restrained/tail-free control and CRF^Hy^ -ablated subjects. By assigning each neuron to a distinct photic responsive class and anatomical area, we compared photic representation at cellular resolution between control and CRF^Hy^ -ablated animals (*n* = 10 per group) (Fig. 6A). Among 165 anatomical areas examined, we uncovered 14 that had significant changes in their photic tuning properties (Fig. 6B–D). The total number of recorded neurons in these anatomical areas were not different between control and CRF^Hy^ neuron-ablated conditions (Fig. S7A, B).

In 11 out of the 14 brain areas, more neurons were tuned to light in CRF^Hy^ -ablated animals (Fig. 6B). These included six diencephalic regions [hypothalamic dopaminergic cluster 6 (#11), eminentia thalami (EmT, #14), hypothalamic hcrtR-cluster (#18 and #19), diencephalic islet1 cluster 3 (#38), left habenula vglut2 cluster (L-dHb, #39)], one mesencephalic region [AF8 (#103)], and four rhombencephalic regions [Gad1b cluster 7 (#152), oculomotor nucleus nIV (#201), olig2-enriched area in the cerebellum (#204), RoL-R1 (#226)]. The evolutionarily conserved retina-EmT-L-dHb pathway is known to regulate light/dark preference [52]. The fact that our unbiased brain-wide analysis uncovers them further validated our analytic methods. Together, these observations suggest that CRF^Hy^ neurons suppress light tuning in these experimentally identified brain areas.

Intriguingly, in 3 out of the 14 brain areas, more neurons were tuned to dark in CRF^Hy^ neuron-ablated animals (Fig. 6C): they are diencephalic isl cluster 1 (#36), rhombencephalic RoM2 (#230) and vglut2 cluster 1 (#247). These observations highlight the complexity of neural circuitry mediating photic perception and the regulation by CRF^Hy^ neurons.

Consistent with the observation that occurrences of struggle, swim, and turn were not different in dark vs. light (Fig. S5G), overall tail movements were not different between control and CRF^Hy^ neuron-ablated subjects (Fig. S7C, D). Neuronal activity associated with vigorous motion was not noticeably different between the two groups (Fig. S7E1, E2). These observations are in line with the previous finding that a rapid defensive behavior measured as fast tail turning in head-restrained tail-free larval zebrafish engage multiple hypothalamic populations with redundant functions [36].

### CRF^Hy^ neurons regulate functional connectivity among selective and distributed brain areas

Brain-wide neuronal activity data at cellular resolution present a salient opportunity to investigate neuronal interactions in an unbiased manner. Functional connectivity (FC) measures correlations between time series of individual neurophysiological events [65], and is not limited to direct anatomical connections. Analysis of FC is particularly relevant in the context of head-restrained tail-free larval zebrafish dataset, since only limited behavioral repertoire can be assessed in this setting. By analyzing FC, it is possible to examine neuronal ensembles that act concertedly in information processing.

**Fig. 7:**
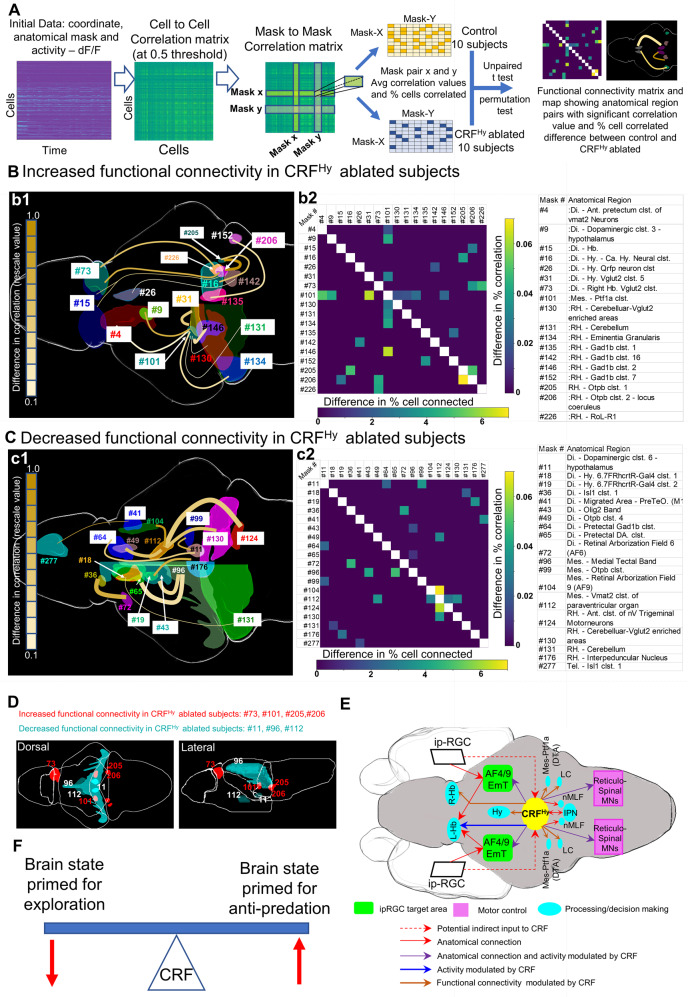
CRF^Hy^ neurons regulate functional connectivity of selective and distributed brain areas. **A** Flow chart showing steps in functional connectivity analysis to identify pairs of anatomical regions showing significant difference between control and CRF^Hy^-ablated subjects. The data carrying activity (dF/F) and anatomical region information for each neuron was used to derive cell to cell correlation matrix with correlation value 0.5 as threshold and the correlation matrix between anatomical regions referred as mask-to-mask correlation matrix. For each anatomical region pair (mask to mask pair) average correlation value and percent cells pairs in correlation were calculated. Comparison of control and CRF^Hy^ ablated (*n* = 10 for each group) was carried out. Mask to mask pair that showed significant increase or decrease in connectivity (both correlation value and percent of significantly correlated cell pairs) were selected to create functional connectivity matrix and to draw the functional connectivity map showing the effect of CRF^Hy^ ablation. **B**, **C** Schematic diagram showing the brain regions with significantly increased (B1) or decreased (C1) functional connectivity in CRF^Hy^-ablated subjects. The color of connecting line represents the rescaled difference in correlation value and the thickness of line represents the rescaled difference in percent of correlated cell pairs. A matrix showing difference in correlation value (right- top) and difference in percent of cells correlated (left -bottom) (B2,C2). The table showing the description for anatomic region ID numbers in B1-B2 and C1-C2. **D** Schematic model describing the circuit controlling the dark avoidance behavior based on the anatomical analysis, comparison of photic response and functional connectivity between control vs CRF^Hy^-ablated subjects. **E** Schematic diagram showing dorsal and lateral views of brain regions that showed significantly increased (red) or decreased (cyan) functional connectivity with at least two other brain regions. **F** Schematic model showing CRF acts as a modulator of the brain state primed for exploration vs anti-predation.

Using the activity of ~14,000 individual neurons per subject as input data, we calculated the neuron-to-neuron connectivity via Pearson correlation as previously described [66], resulting in ~225 million neuronal pairs per subject. We next calculated neuroanatomical mask-to-mask connectivity. A total of 135 anatomical masks and 9,045 mask pairs were examined. For each mask pair, both Pearson correlation values and number of neuronal pairs in the masks above the threshold value (0.5) were computed for control and CRF^Hy^ -ablated animals (*n* = 10 per group) (Fig. 7A).

Through such analyses, we found 14 mask pairs with increased FC in CRF^Hy^ -ablated subjects (Fig. 7B, D, and Table S1). Strikingly, seven out of 14 pairs involved the mask “Mes. -Ptf1a” (#101), which represented ~15 neurons in our recorded dataset. These neurons, likely derived from the upper rhombic lip progenitors expressing the proneural gene *ptf1a* [67], are in the dorsal tegmental area (DTA) bordering the cerebellum. Its FC to diencephalic (#4, #9, #31) and hindbrain areas (#130, #131, #134, #146) were increased in CRF^Hy^ -ablated subjects. Interestingly, DTA, which is homologous to mammalian periaqueductal gray (PAG), dorsal tegmental nucleus and nucleus incertus, is known to receive projections from the IPN [68]. IPN is directly innervated by the L-dHb, which, as described earlier, receives retina-EmT input [52]. Other anatomical areas worth noting are the hindbrain locus coeruleus (LC) (#206), whose FC to one hindbrain (#205) and two diencephalic areas (#15, #73) was increased; the right habenula vglut2 cluster (#73) with increased FC to two hindbrain areas (#226 and #206), and the rhombencephalic Otpb cluster 1 (#205) with increased FC to one diencephalic area (#16) and the hindbrain LC (#206).

Twelve mask pairs showed decreased FC in CRF^Hy^ -ablated conditions (Fig. 7C, D, and Table S2). Four out of twelve involved the anatomical mask named “Mes. -Vmat2 clst. of paraventricular organ” (#112), which has ~27 neurons in our recorded dataset and are in the ventral tegmental area close to the diencephalon. Its FC to one diencephalic region (#49), one midbrain region (#104), and two hindbrain regions (#124 and #130) was significantly decreased. Other anatomical regions worth noting were Mes. -medial tectal band (#96) and Di DA clst. 6 (#11), whose FC to two anatomical areas were decreased. Together, CRF^Hy^ neurons critically regulate the FC of distributed brain areas.

## Discussion

Here we investigate brain-wide perception of the emotional valence of light and show that these processes are regulated by distinct hypothalamic neuronal types in larval zebrafish. We demonstrate both a necessity and a sufficiency of CRF^Hy^ neurons in promoting avoidance of the space that activate these neurons, including the dark. We further show that individual CRF^Hy^ neurons extend processes to diverse brain areas including sensory, motor, and decision-making, and their activity is regulated by the light/dark stimuli in a complex way, with the overall sum of activity being reduced by light. Brain-wide calcium imaging during alternating phases of light/dark stimuli reveal distributed coding of photic features that are regulated by CRF^Hy^ neurons in selected anatomical clusters. Overall, our study illustrates the brain-wide impact of alternating light/dark photic stimuli at cellular resolution. It uncovers, for the first time, the role of CRF^Hy^ neurons in the perception and emotional valence of light as well as in regulating brain-wide functional connectivity. It also provides a plausible mechanism for the effectiveness of light in treating mood disorders.

### Role of hypothalamic peptidergic neurons in mediating the emotional valence of light

Photic stimuli carry distinct emotional valence information in diverse species across their life stages [7, 43, 69]. Larval zebrafish display positive phototaxis, a short-burst behavior (on the order of seconds) that actively orients the animals toward a light source and involves stereo-visual comparison [70], spatiotemporal sampling [71], and a self-oscillating hindbrain population (HBO) [72]. Positive phototaxis suggests that light carries a positive valence (e.g., associated with warmth or food) whereas dark has a negative valence (e.g., associated with coldness or shadows of predators).

The light/dark preference behavior, measured on the order of minutes and assessing total time spent in dark vs. light territories, engages more complex patterns of neuromodulation and a process of “decision-making” super-imposed onto a phototactic reflex. The molecular and cellular mechanisms underlying this preference behavior are only beginning to be understood. Kinematic analyses of behavior uncovered that CRF^Hy^ neurons regulate a decision at the border, resulting in a decreased probability of entering the space where CRF^Hy^ neurons become activated. Pharmacological inhibition of CRF signaling and genetic knockout of the *crhb* gene established the importance of neuropeptide signaling. This contrasts with a previous observation, which shows that a fast-timescale (on the order of seconds) defensive behavioral response in head-restrained/tail-free larval zebrafish is dependent on glutamate co-released from multiple sets of hypothalamic peptidergic neurons [36]. Our findings of CRF^Hy^ neurons promoting whereas neighboring oxytocin neurons suppressing dark avoidance indicate that distinct hypothalamic peptidergic neurons have distinct effects on the light/dark preference behavior. Future mechanistic studies of oxytocin neurons’ role in decreasing dark avoidance shall shed new light on the behavior and involvement of these neurons, which are recently shown to regulate defensive responses to noxious input [55].

### Breadth and heterogeneity of CRF^Hy^ neuron projections revealed by single-cell imaging in an intact vertebrate brain

Neuronal morphology is a distinguishing feature of cell type and associated functionality [73]. Larval zebrafish, with its small brain containing few cells per neuronal class (estimated to be ~1000 fold less than the mammalian brains) [74], offers an excellent opportunity to classify single-neuron projection patterns in an intact vertebrate brain. Here, we applied such analysis to the genetically accessible CRF^Hy^ neurons, estimated to be a total of ~28 (±3) in the larval Int-Hy and ~50 (±3) in Po, PT, and Int-Hy combined. Previous bulk labeling has shed light on the distribution and morphological features of CRF neurons in vertebrate brains [60, 75], but these studies are not able to discern the breadth of each neuron’s projection.

CRF^Hy^ neurons are classically thought to only regulate physiological stress response, whereas another CRF neuronal population in the lateral central nucleus of the amygdala (CeA_L_) is extensively studied in the context of conditioned fear [76–78]. Recent studies, however, have uncovered a crucial role of CRF^Hy^ neurons in innate fear-related behaviors, in both mice [23–25, 79] and zebrafish (this study). Consistent with this notion, we observed CRF^Hy^ neuronal processes and varicosities in behaviorally relevant brain areas, including non-image forming visual fields (e.g., AF4, AF9), pre-motor and motor (e.g., nMLF, Rol1-R1), and decision-making areas (e.g., IPN, and DA clusters). Moreover, single cell analysis of 34 individual (representing over 60% of genetically accessible) CRF^Hy^ neurons uncovered two previously unknown features: First, a single CRF^Hy^ neuron can send processes to as many as ten brain regions, both ipsi- and contra-laterally, demonstrating that broad connectivity is an individual rather than a group feature. Second, heterogeneity exists even within an anatomically clustered group of neurons, with some having more elaborate processes whereas others having relatively few. This morphological heterogeneity likely underlies their different photic response properties observed in our calcium imaging data. However, functionally classified CRF neurons were not found to be in defined anatomical locations across the recorded subjects, thereby precluding the possibility to correlate projection patterns with functional classifications using different subjects. Future work to record morphology, neural activity, and gene expression profiles in the same cell shall provide further insights into cell type classification and structure-function relationships.

### Brain-wide photic perception through distinct, distributed, and tunable neuronal response types

Significant advances have been made in understanding how physiochemical stimuli such as light, sound, odorants, and temperature/touch are transformed into electrical impulses in sensory neurons, but how these stimuli are perceived by the brain remains poorly understood. Photic perception is an organism’s ability to perceive the surrounding light or dark information. While brain-wide activity in response to patterned visual stimuli have been observed [28, 80], how light dark information is represented in the brain has not been systematically analyzed. Other tissues such as pineal or preoptic area are photosensitive [81] in addition to retina. Since light/dark preference behavior is largely abolished in retinal RGC-deficient larval zebrafish, RGCs are the most prominent cell types that transduce photic information to the brain. By brain-wide calcium imaging upon alternating light or dark stimuli, we were able to classify most neurons in the brain into distinct photic response types, including those repeatedly activated in light or dark, those activated during first periods of light or dark, and those transiently activated during light–dark transitions. Several features of photic perception are worth noting. First, distinct photic response types are distributed in an intermingled manner in the brain. Most photic response types are likely present in most anatomical areas. Second, photic response types are functionally rather than anatomically defined, as the response types are tunable brain-wide (e.g., by CRF^Hy^ neurons). Third, response types that are “activated in light” or “activated in first dark period” are also robustly activated by vigorous motion, more so than other photic response types. How photic perception as described in this study is established in the brain is an important question for future investigation. Neurons activated at light–dark transitions are notably few in numbers and may represent a good entry point for further research.

### A model depicting the role of CRF^Hy^ neurons in regulating photic perception and brain state

To uncover brain-wide photic perception at cellular resolution, we needed to use head-restrained animals. One limitation with such preparation is the impact of restraining stress. At the behavioral level, we observed periodic, vigorous tail movements that are accompanied by robust brain-wide neuronal activation. The frequency of vigorous motion was not different in light vs dark, suggesting head restraining, a much stronger threat, can mask the valence of photic stimuli in behavioral expression. Despite this, brain-wide activity tuned to photic stimuli were still robustly observed.

Disruption of CRF^Hy^ neurons did not alter the frequency of vigorous motion in head-restrained subjects. This is consistent with previous observations in mice [76] and in zebrafish [36], which suggest that strong threats likely engage broader neuronal groups than CRF alone. However, disruption of CRF^Hy^ neurons did alter photic tuning in selected brain areas, with most (11/14) increasing light tuning, *that is*, neurons activated in light are increased at the expense of those activated in dark, suggesting a switch of photic tuning properties. Thus, CRF^Hy^ neurons suppress neural representation of light in the brain. By integrating our anatomical and neural activity data with previous work [52], we propose the following circuit model for the light/dark preference behavior (Fig. 7E): Light activates the melanopsin-expressing ipRGCs, which project to the AF4 and AF9 areas (non-image forming visual fields), to activate L-dHb via EmT. Habenula then connects, either directly or indirectly via IPN-DTA and nMLF, to the reticulospinal motor neurons to control action selection (i.e., approach light). Dark-activated CRF^Hy^ neurons further intersect this phototactic circuit at multiple levels, including AF4 and AF9, nMLF, IPN, and finally, reticulospinal motor neurons.

CRF^Hy^ neurons receive input from many different brain areas in mammals [82], and play an integral role in response to threat [79] that is not limited to light/dark stimuli. Built on this concept, we performed brain-wide FC analysis at cellular resolution followed by grouping within anatomical areas to unbiasedly discover those with altered FC when CRF^Hy^ neurons are ablated. It is worth noting that, while functional cell types are often organized into compact brain nuclei, anatomical areas do not always correspond to homogeneous functional cell clusters. Despite this limitation, we identified FCs between certain anatomical areas that were significantly altered by removing CRF^Hy^ neurons. Of great interests are the mesencephalic Ptf1a cluster and rhombencephalic Otpb cluster 2–locus coeruleus, whose FC to 7 or 3 brain areas respectively were increased in CRF^Hy^-ablated subjects. While little functional data are available for the mesencephalic Ptf1a cluster, it is located in the DTA bordering the cerebellum, which receives inputs from IPN, a target of habenula [68]. Locus coeruleus is well known for promoting arousal [83–85]. Conversely, the mesencephalic vmat2 cluster of paraventricular organ and multiple hypothalamic neuronal clusters were among those with decreased FC in CRF^Hy^-ablated subjects. While little is known about the function of vmat2 cluster, several hypothalamic neuronal clusters are involved in stress/fear responses [86]. Therefore, it is tantalizing to hypothesize that CRF^Hy^ neurons regulate a transition between brain states of stress/threat-free exploration and stress/threat-triggered anti-predation; in the absence of CRF^Hy^ neurons, the balance is tipped to favor exploration at the expense of anti-predation (Fig. 7F). Such brain states may not impact behavior, or alternatively, can drive a variety of different behaviors in a context-dependent manner [79].

### Relationships between perception, brain state, and behavior

Perception is the sensory experience of the world. Our experimental data show that it is possible to perceive (as evidenced by altered brain state) without altering the limited behavioral output measurable under the head-restrained condition. According to the evidence accumulation model [87, 88], animals need to integrate diverse sensory stimuli, evaluate, and decide on whether and when a behavioral response is warranted. The process of choosing whether a behavior is produced and when it is produced, is an active area of investigation in various organisms [31, 89–92].

In head-restrained/tail-free larval zebrafish, where both brain-wide neuronal activity and tail movement behavior can be recorded upon delivery of light/dark stimuli, we observed robust photic perception, but the probability of tail movement behavior was not different in light or in dark. Similarly, in CRF^Hy^-ablated subjects, we observed altered neural activity and functional connectivity in discrete brain areas, but the probability of tail movement behavior was unaffected. Vigorous motion is accompanied by widespread neuronal activation. Extinction of vigorous motion (or giving up) involves astroglia activity and noradrenaline [30], but what generates vigorous motion is unclear. It has been observed that hypothalamic outputs of glutamatergic signals from multiple overlapping peptidergic neurons converge on brainstem neurons to drive vigorous tail turns [36]. We found that the top 1,000 neurons activated during vigorous motion were distributed across brain areas, suggesting a distributed property of the underlying circuit.

### Evolutionary perspective of light, CRF^Hy^, and mood disorders

Several lines of evidence suggest that the effect of photic stimuli on brain state and behavior is evolutionarily conserved across vertebrates. Previous findings of the ipRGC-EmT-L-dHb involvement in light preference in zebrafish [52] and the ipRGC-PHb in light-mediated mood alterations in mice [5] highlight the importance of retinal melanopsin neurons and habenula. Our work further implicated the IPN-DTA pathway downstream of Hb as well as the pre-motor-motor areas (nMLF-Reticulospinal MNs). IPN is a phylogenetically conserved brain area that integrates information for the limbic system [93]. In mammals, the medial habenula (homologous to the dorsal habenula in zebrafish)-IPN circuitry is critical for addiction, anxiety, and mood regulation [94]. In zebrafish, IPN is involved in sensorimotor decision-making [92]. Downstream of the IPN is the DTA, which contains the mesencephalic pdf1a cluster, whose FC with seven brain areas was significantly enhanced by removing CRF^Hy^ neurons. DTA is homologous to the mammalian periaqueductal gray (PAG), which is implicated in behaviors related to fear or stress [68].

Last but not the least, a recent study in rodents showed that CRF^Hy^ neurons in the preoptic area receive ipRGC input [95], suggesting that the role of CRF^Hy^ neurons in regulating the perception and valence of light may also be conserved across species. In conjunction with the observed effectiveness of light therapy for treating mood disorders in humans [2–4], we propose that suppression of CRF^Hy^ neuronal activity is a plausible mechanism by which light elevates mood.

## Supplementary information

S1

S2

S3

S4

Supplemental material

## Data Availability

All primary data are stored on a secure server at the University of California, San Francisco and are available from the corresponding author.

## Code availability

Full coding implementation of all analysis tools are available at https://github.com/Mahdizarei/Brain-wide-perception-of-the-emotional-valence-of-light-is-regulated-by-distinct-hypothalamic-neuron.
