# Correction to: Brain-wide perception of the emotional valence of light is regulated by distinct hypothalamic neurons

**DOI:** 10.1038/s41380-022-01647-y

**Published:** 2022-06-20

**Authors:** Mahendra Wagle, Mahdi Zarei, Matthew Lovett-Barron, Kristina Tyler Poston, Jin Xu, Vince Ramey, Katherine S. Pollard, David A. Prober, Jay Schulkin, Karl Deisseroth, Su Guo

**Affiliations:** 1 Department of Bioengineering and Therapeutic Sciences, University of California, San Francisco, CA 94143-2811 USA; 2 Department of Bioengineering, Howard Hughes Medical Institute, Stanford University, Stanford, CA USA; 3 Tianqiao and Chrissy Chen Institute for Neuroscience, Division of Biology and Biological Engineering, California Institute of Technology, Pasadena, CA 91125 USA; 4 Biophysics Graduate Group, University of California, Berkeley, CA USA; 5 Gladstone Institute of Data Science & Biotechnology, San Francisco, CA USA; 6 Department of Epidemiology & Biostatistics, University of California, San Francisco, CA USA; 7 Chan Zuckerberg Biohub, San Francisco, CA USA; 8 Department of Obstetrics & Gynecology, School of Medicine, University of Washington, Seattle, WA USA; 9 Programs in Human Genetics and Biological Sciences, Kavli Institute of Fundamental Neuroscience, The Eli and Edythe Broad Center of Regeneration Medicine and Stem Cell Research, Bakar Aging Research Institute, University of California, San Francisco, CA 94143-2811 USA; 10 Present Address: Neurobiology Section, Division of Biological Sciences, University of California, San Diego, La Jolla, CA USA; 11 Present Address: Invitae Inc., San Francisco, CA USA

**Keywords:** Neuroscience, Genetics

Correction to: *Molecular Psychiatry* 10.1038/s41380-022-01567-x, published online 28 April 2022

The wrong Supplementary file was originally published with this article; it has now been replaced with the correct file, and referenced appropriately in the main text.

The original article has been corrected.

