# Supplementary material for: Brain-wide perception of the emotional valence of light is regulated by distinct hypothalamic neurons
Source: Mol Psychiatry. 2022 Apr 28;27(9):3777–93. doi: 10.1038/s41380-022-01567-x (PMC9613822; doi:10.1038/s41380-022-01567-x)

KEY RESOURCES TABLE

REAGENT OR RESOURCE	SOURCE	IDENTIFIER
Chemicals and antibodies		
Metranidazole	Sigma Aldrich	M3761 CAS #: 443-48-1
Antalarmin	Sigma Aldrich	A8727 CAS #: 220953-69-5
Mivacurium chloride	Abcam	ab143667 CAS #: 106861-44-3
Lipophilic dye DiD	ThermoFisher	V22887 CAS # : 127274-91-3
Alexa-568 conjugated Dextran -10Kd	ThermoFisher	D22912
Tubocurarine	Sigma-Aldrich	T2379, CAS # 6989-98-6
anti-GFP antibodies	Abcam	RRID:AB_300798
anti-ACTH antibodies	National Hormone & Peptide Program, Torrance, CA	RRID:AB_2313902
anti-tyrosine hydroxylase antibodies	Sigma-Aldrich	RRID:AB_390204
anti-5HT antibodies	Immunostar	RRID:AB_572263
anit-chicken Alexa 488 antibodies	ThermoFisher	A-11039
anti-rabbit Alexa 568 antibodies	ThermoFisher	A-11036
Primers		
Genotype: crhb For	Integrated DNA Technologies	5'-CCGAGACATCCCAGTATCCA-3'
Genotype: crhb Rev	Integrated DNA Technologies	5'-TGGCCATCTCCAGTACTTCTC-3'
Plasmids		
pmTol2-crf:Gal4FF	This paper	SG1347_13-2
UAS:Synaptophysin-RFP	[1]	p362 [tol1] 14xUAS-Synaptophysin-TagRFPT
pmTol2-UAS:PSD95-GFP	[2] and This paper	
Animal, Transgenic		
Zebrafish: Tg(elavl3:H2BGCaMP6s)	[3]	JF5
Zebrafish: Tg(crf:Gal4FF)	[4]and this paper	*s2510*
Zebrafish: Tg(pomc:Gal4FF)	[4] and this paper	*s2511*
Zebrafish: Tg(oxt:Gal4)	[5]	N/A
Zebrafish: Tg(sst3:Gal4)	[6]	Mpn219
Zebrafish: Tg(otpba:Gal4)	[7]	Zc57
Zebrafish: Tg(UAS:GFP)	[8]	Nns19
Zebrafish: Tg(UAS:ChR2-mCherry)	[9]	Mpn134
Zebrafish: Tg(UAS:NTR-mCherry)	[10]	Rw0144
Animal, mutant		
crhb	This study	*ct862*

Software		
Noldus Ethovision XT	Noldus	https://www.noldus.com/ethovision-xt
Imaris	Oxford Instruments	https://imaris.oxinst.com/
Fiji (image J)	[11]	http://fiji.sc/
CMTK	[12]	https://www.nitrc.org/projects/cmtk/
CaIMaN	[13]	https://github.com/flatironinstitute/CaImAn
Python 3.5	Python	https://www.python.org/
Numpy	[14]	https://www.numpy.org/
Scipy	[15]	https://scipy.org/
Scikit Image 0.15.0	[16]	https://scikit-image.org/
Scikit Learn 0.17.0	[17]	http://scikit-learn.org/
Statsmodels 0.8.0	[18]	http://www.statsmodels.org/
Matplotlib 3.1.1	[19]	https://matplotlib.org/
Heapq	Python Doc	https://docs.python.org/3/library/heapq.html
Seaborn 0.9.0	Seaborn	https://seaborn.pydata.org/
Bokeh 1.2.0	Bokeh Development Team	https://bokeh.pydata.org/en/latest/
Jupyter	[20]	https://jupyter.org/
Pandas	[21]	https://pandas.pydata.org/

Supplementary Methods

Plasmid constructs and the crf transgenic line establishment

Although the *Tg[crf-gal4FF]* was previously validated and used in our study [4], detailed methodology on how it was generated warrants description here. Conserved elements from the *crhb* locus were identified via UCSC Genome Browser conservation tacks (UCSC Genome Browser on Zebrafish July 2007 Zv7/danRer5) based on multi-species sequence alignments [22]. These elements were amplified using the PCR primers listed in Fig. S1B and digested with restriction enzymes to generate sticky ends. Fragments were randomly ligated with pMiniTol2 vector [23] upstream of tdTOM. Resulting clones were checked for insert by restriction enzyme digest. Clones with more than 1000 bp inserts were microinjected in zebrafish embryos and screened for neuronal expression 48 hpf onwards. Total 28 clones were tested. The expression was compared with endogenous *crhb* expression. One of the clones (13-2), which contained Enhancer 5, Enhancer 2 (partial), and Enhancer 1, showed expression in the hypothalamus similar to endogenous *crhb* expression. For generating CRF^{Hy} transgenic lines, tdTOM was replaced with Gal4FF and injected in UAS-GFP transgenic embryos and raised to adulthood. The F0 fish were crossed with UAS-GFP to identify the transgenic carriers. Two F0 founders (out of 30 screened) showing identical expression were used to establish the CRF^{Hy} transgenic line. The UAS reporter lines were generated by crossing CRF^{Hy}-gal4FF with corresponding UAS lines.

Plasmid constructs and the pomc transgenic line establishment

The 1080 bp fragment containing 1029 bp *pomc* promoter fragment was amplified from the *pomc*-GFP construct (a gift from Dr. Ning-Ai Liu)[24] using the vector primer 5'- CCCCCTCGAGGTCGACG-3' and *pomc* primer upstream of ATG: 5'-ATCCTCACTCCCCTCACCAT-3'. The PCR product was cloned upstream of Gal4 in pminiTo2 vector (a gift from Stephen Ekker, Addgene plasmid # 31829; http://n2t.net/addgene:31829; RRID: Addgene_31829)[23]. The resultant construct was injected into transgenic embryos carrying UAS-GFP to establish *Tg[pomc:gal4FF].*

Cortisol extraction

Cortisol extraction and analysis performed as previously described [25]. For each treatment group, 6 replicates (each carrying 10 larvae) were used for cortisol extraction. Cortisol levels were normalized to total protein and plotted as ratio of non-stressed vs stressed within each treatment group.

Neuronal ablation

Gal4 transgenic lines were crossed with UAS::NTR-mCherry [10] to generate transgenic larvae expressing UAS::NTR-mCherry in specific neuronal subtypes. Transgenic and non-transgenic larvae were screened and separated at 3 dpf and incubated at 28 ^{0}C with 14-10 hours light/dark cycle. Larvae were treated with metronidazole (4.5 mM or 9.0 mM MTZ) or 0.2% DMSO from 4dpf to 6dpf with daily change of embryo medium. At 6dpf, larvae were transferred to E3 medium and behavioral recording was carried out on 7 dpf.

Immunostaining

Transgenic larvae expressing GFP in CRF^{Hy} neurons were treated with 0.003% PTU from 24hpf onwards and fixed with 4% PFA at 5dpf. Larvae were treated with methanol and rehydrated in PBT (PBS with 0.5% Triton X-100). Larval brain was exposed by removing eyes and jaw cartilage. Dissected larvae were blocked in blocking solution (5% normal goat sue rum in PBT) at room temperature for 3-4 hours and then incubated with primary antibodies, Anti-TH at 1:1000 dilution, or Anti-5HT 1:10000 dilution, and Anti-GFP 1:1000 dilution for 3 days at 4 ^{0}C. Larvae were washed 8 times with PBT at room temperature and incubated with secondary antibody anti-chicken-Alexa 488 and anti-rabbit-Alex568 at 1:500 dilution overnight at 4 ^{0}C. Immunostained larvae were washed 5 times with PBT and mounted in 70% glycerol in PBS for imaging.

In vivo recording of CRF^{Hy} channelrhodopsin-expressing neurons

Neurons were recorded from 5 d.p.f. Tg(crf:Gal4FF; UAS:ChR2-mCherry) larvae. Larval zebrafish were anesthetized in 0.1% MS-222 (Sigma) and paralyzed with 5mM tubocurarine (Sigma-Aldrich T2379) diluted in extracellular recording solution (134 mM NaCl, 2.9 mM KCl, 2.1 mM CaCl2, 1.2 mM MgCl2, 10 mM glucose, 10 mM HEPES, pH7.8). Fish were mounted on their side, and mCherry+ neurons were visually identified using an upright microscope (Olympus BX51WI) and CCD camera (Q-Imaging RoleraXR). Recording pipettes with 9–12-MΩ tip resistance were used, filled with external solution. After reaching a ≥1-GΩ seal, the internal

membrane was not ruptured to maintain the cell attached configuration (voltage clamp, no holding voltage). Light was delivered with a Spectra X LED light engine (Lumencor) via a 475/35 nm filter and ×40/0.8 NA water-immersion objective (3 mW power measured from objective). Light stimulation was applied to neurons with spontaneous spiking at baseline (10 ms pulses separated by 1 s, a 3 s pause, and 10 s of 100 ms pulses at 5 Hz).

Behavior

---Camouflage assay

Camouflage assay was performed as previously described [26]. For each group 16 larvae were used.

---Light dark preference behavior

Light dark preference behavior was performed as previously described [25]. Larvae were raised at 28 ^{0}C with 14-10 hours light/dark cycle on light blue background. All behavior recording was performed with 7 dpf larvae between 9.00 AM to 4.00 PM. Larvae were transferred to behavior recording room and left on light blue pad for one hour. Recording was performed in batches of 8 larvae per experimental group. Each behavior chamber had the dimension of 4 cm x 4 cm x 1 cm, with walls covered with white and black tapes (Fig 1A). All 8 larvae were pipetted into the center of the behavior recording chambers kept on the blue background. All chambers with larvae were transferred on to the recording stage made by covering the white light transilluminator with clear transparent and black (infrared transparent) acrylic strips such that each chamber is divided into light and dark zone from the bottom matching with the tape color on the walls of chamber. Recording was performed with a Panasonic CCD camera fitted with the IR filter connected to computer. Four chambers were recorded simultaneously from a single camera with Noldus Ethovision Recorder. Recording was captured for 8 minutes at 30fps and stored in MPEG4 format for further analysis with Noldus Ethovision XT software.

For CRF receptor antagonist, AB-wildtype larvae were raised at 28 ^{0}C with 14-10 hours light/dark cycle from 3 dpf to 7 dpf in E3 medium. Larvae were incubated with 1uM Antalarmin (CRFR1 antagonist) or 0.2% DMSO in E3 medium for one hour. Larval behavior was recorded in E3 buffer with Antalarmin.

The *crhb*$^{-/-}$ CRISPR knockout and sibling larvae were obtained by crossing heterozygous *crhb*$^{+/-}$ adults and raising at 28 ^{0}C with 14-10 hours light/dark cycle from 3 dpf to 7 dpf in E3 medium. Larvae were genotyped individually after behavior recording and grouped accordingly for analysis. Genoyping PCR with primer flanking the indel site showed two bands in with heterozygous larvae corresponding to homoduplex and heteroduplex. The second round PCR was performed by mixing the wildtype DNA to differentiate *crhb*$^{-/-}$ mutant vs *crhb*$^{+/+}$ wildtype. The two round CPR method was validated by sequencing the CPR product (Fig. S3).

---Behavioral preference upon optogenetic stimulation

CRF^{Hy}:Gal4FF; UAS::GFP transgenic line was crossed with UAS:: ChR2-mCherry [9] to generate CRF^{Hy}::Gal4FF; UAS:: ChR2-mCherry transgenic line. Embryos obtained from in-cross of transgenic CRF^{Hy}:Gal4FF; UAS::ChR2-mCherry heterozygous, were screened for mCherry expression at 3 dpf under stereo florescence microscope. Transgenic larvae (mCherry positive) and non-transgenic (mCherry negative) were separately incubated at 28 ^{0}C with 14-10 hours light/dark cycle on grey metallic background and transferred to behavior recording room at 7 dpf. Behavior chambers were similar to the light/dark choice behavior chamber with side wall covered with white tape. Larvae were individually transferred to 8 behavior chambers on grey metallic background. The recording stage was modified for optogenetic behavior by placing a 9 cm(L) x 9 cm (W)x 6.5 cm(H) box on a transilluminator with center compartment of 9 cm(L) x 4.5 cm(W) carrying five Blue LEDs (Chanzon 3 W Royal Blue 440 nm-450 nm / 400 mA-500 mA / DC 3 V-3.4 V / 3 Watt) mounted on a heat sink (Chanzon 1 W 3 W 5 W LED Heat Sink, 2 pin White, Aluminum Base Plate). The top of the box was covered with clear acrylic and a diffuser (Rosco Cinegel Tough Rolux) and blue filter (Lee Filters Bright Blue -Gel filter sheet) (Fig. 4A). Set of 4 chambers were placed on top of the box such that each chamber is half illuminated with white light from transilluminator and the other half with blue LEDs. The distance between blue LEDs and the chamber was 5 cm allowing 10 mW/cm^2 of blue light at the chamber (recorded with Newport optical power meter 1916-C). The behavior recording was performed as above.

In vivo labeling and imaging

---Single neuron labeling

The crf::GAL4FF and UAS::tdTomato plasmid constructs were injected at 60 ng/ul (4-6 nl/embryo) into Tg[HuC::H2B-GCAMP6s] transgenic line at 1-4 cell stage to sparsely label CRF^{Hy} neurons. Embryos were screened at 3-4 dpf under a stereo florescence microscope for neuronal expression of tdTomato. At 7 dpf larvae were imaged under a confocal microscope. The image stack from each larva was registered with Z-brain Elavl3-H2BRFP template and compared with our reference CRF^{Hy} transgenic brain to identify the position of labelled cell soma and areas of neuronal projections. Anatomical masks from offline Z-brain were overlapped in imageJ.

---Dye labeling

To label the optic tract and spinal projection neurons, 6 dpf larvae were anaesthetized with MS222 and embedded in the low melting agarose. Lipophilic dye DiD (ThermoFisher-V22887) solution at 2 mg/ml in ethanol was injected into the eye of 6dpf larva to fill the retinal layer. Alexa-568 conjugated Dextran (10KD, ThermoFisher -D22912) at 30% in Deneau’s solution was injected into the spinal cord just behind the cloaca from the dorsal side. Larvae were rescued from agarose and allowed to recover and for the dye to spread for at least 3 hours. Labeled larvae were embedded again in low melting agarose for imaging on a Zeiss LSM 780 confocal microscope. Images were processed with Imaris for tracing neuronal projections.

---In vivo calcium imaging

For imaging CRF^{Hy} neuronal activity, CRF^{Hy}::Gal4FF; UAS::NTR-mCherry fish were crossed with UAS::GCaMP6s to generate CRF^{Hy}::Gal4FF; UAS::GCaMP6s. At 6 dpf larvae were paralyzed in a drop of neuromuscular-blocking drug mivacurium chloride (1mg/ml) and mounted on the glass bottom holder in 1.5% low melting point agarose, covered with 1.5 ml E3 medium and placed on temperature-controlled stage at 24^0C. The 2P excitation was set at 920 nm, 40 um volume was imaged with 20x objective controlled by piezo keeping 10-micron step size to cover most of transgenic crf neurons expressing GCaMP6s within the intermediate hypothalamus. Imaging was carried out at 2 Hz at 780 x 780-pixel resolution (0.887 microns/pixel). Light stimuli was delivered at 200 frames on/off intervals (corresponding to 100 sec based on 2 Hz acquisition) with 455 nm fiber coupled LED (Thorlabs – M455F3) controlled with T-cube Led driver (Thorlabs-LEDD1B). The optical fiber end was placed above the larva at approximately 5 cm away and at 45-degree angle with spot lens (uxcell) attached. The light intensity at the imaging stage was 3 mW/cm^2. Within the Light-ON duration of 100 sec, LED pulses were delivered at 40Hz to intercalate between the frames to avoid bleed through into PMT.

For simultaneous CRF^{Hy} neuronal activity imaging and tail swing recording, non-paralyzed larvae were mounted on the lid of 35 mm petri dish in 70 ul of 2% low melting agarose. The tail was set free by removing agarose covering the trunk below the otic vesicles and covered with 3.5 ml E3. Imaging was carried out on 3i VIVO set up as above with addition of sub-stage camera (FLIR CM3-U3-13Y3M-CS monochrome). Larvae were illuminated with 800 nm infra-red LEDs (Phenas Home). The 700 nm cut-on and 850 nm cut-off filters were placed in path to block blue light form stimulating LED and 920 nm imaging laser. The tail movement was recorded at 100 fps through a sub-stage camera. Light stimuli were delivered at 100 frames on/off intervals (corresponding to 100 sec based on 1 Hz acquisition)

For brain wide calcium imaging, HuC::H2B-GCaMP6s transgenic fish were crossed with CRF^{Hy}::GFF; UAS::NTR-mCherry; UAS::GCaMP6s. Larvae were screened for all transgenes at 3 dpf. For CRF^{Hy} ablation, larvae were treated with MTZ as described above. Neuronal activity imaging and tail swing recording was carried out on 6-7 dpf larvae as described above. The brain wide imaging was carried out for anterior brain (covering forebrain- midbrain and part of hindbrain) covering 504 x 484 microns field of view with zeiss 20x objective. Each volume was 200 microns covered in 11 z-planes with 20 microns step size. The imaging was carried out at 1 Hz with 390 x380 pixel resolution (1.273 microns/pixel). The tail movement was recorded at 100 fps through sub-stage camera.

Data processing

---Image registration

Images z-stack files were saved in the nrrd format. For generating the reference CRF^{Hy} transgenic brain, one of the stacks was used as the template to which image stacks from 9 individual larvae were registered. The reference CRF^{Hy} transgenic brain was generated by averaging the 10 registered stacks. The reference CRF^{Hy} transgenic brain was registered to Elavl3-H2BRFP stack from Z-brain atlas. We used CMTK on local cluster

computing (UCSF Wynton) and passed on the parameters -a -w -r 010203 -X 104 -C 8 -G 160 -R 2 -J 0.050 -A '--dofs 6,9 --mi --accuracy 8' -W '--mi --accuracy 8' -v -T 4 -s. The warp coefficient generated by CMTK were used with CMTK reformat for image stack transformation. The resultant stack was compared with Elavl3-H2BRFP in ImageJ. Image stacks with anatomical masks were downloaded from offline Zbrain viewer and merged on to reformatted image stacks in ImageJ.

---Neuronal activity data processing

Brain-wide neuronal activity image stacks containing time series from each Z-plane were processed with CaImAn [13] using the following parameters.

```
# Parameters for source extraction and deconvolution
p = 1                        # order of the autoregressive system
gnb = 2                      # number of global background components
merge_thr = 1                # merging threshold, max correlation allowed
rf = 5                       # half-size of the patches in pixels. e.g., if rf=25, patches are 50x50
stride_cnmf = 2              # amount of overlap between the patches in pixels
K = 8                        # number of components per patch
gSig = [2, 2]                # expected half size of neurons in pixels
method_init = 'greedy_roi' # initialization method (if analyzing dendritic data using 'sparse_nmf')
ssub = 1                     # spatial subsampling during initialization
tsub = 1                     # temporal subsampling during intialization
# Parameters for component evaluation
min_SNR = 2                   # signal to noise ratio for accepting a component
rval_thr = 0.9                # space correlation threshold for accepting a component
cnn_thr = 0.1                 # threshold for CNN based classifier
cnn_lowest = 0.2              # neurons with cnn probability lower than this value are rejected
```

Output files carries the coordinate and dff (GCaMP fluorescence variation) for each extracted ROI. For brain wide imaging around 24000 regions of interest (ROIs) were extracted from 11 z-panes for each individual larva. These outputs from CaImAn were processed through custom written script to assign Z-brain anatomical masks for each ROI. The false positive ROIs detected outside the brain and those corresponding to anatomical mask 'Ganglia – Eyes’ were eliminated for further analysis. Thus, the final output carried coordinate, dff and anatomical masks for each ROI.

---Tail movement behavioral data processing

Tail swing recording videos were processed using a Matlab script [27] to extract tip deflection angle at each frame. The tail base point was assigned posterior to swim bladder and the tail tip was assigned slightly anterior to the visible end of the tail. The tail tip angle plot data was exported to excel files for further analysis.

Data Analysis

---Classification of cells (ROIs) based on their photic response properties

The cells were classified based on their activity correlation with photic stimuli using custom written script. The algorithm for classification of a cell based on the light/dark response is as follows. In the first step of initialization the light time windows T_{light} and dark time windows T_{dark} during whole time interval T were defined. For a given cell with signal δ $(\Delta F/F)$, $\delta^{all}_{stimulus,i}$ $\delta^{qr}_{stimulus,i}$, and $\delta^{1th}_{stimulus,i}$ denotes calcium variation during ith stimuli window, quarter of the ith stimuli window and the first time window for a stimuli respectively. Let $\delta^{nlargest}$ be the n largest signal value of the signal and δ_{avg} the calcium traces average value across time.

Next cells are classified in to six classes - light responsive, dark responsive, responsive in transition from dark to light, responsive in transition from light to dark, active in first light phase, and active in first dark phase as follows.

Define the tw_{light} and tw_{dark}, where

$$tw^{all}_{light} = \{l: l \in T_{light}\ and\ averag\ (\delta^{nlargest}_{light,l}\ ) > \ \delta_{avg}\},$$

$$tw^{all}_{dark} = \{l: l \in T_{light}\ and\ averag\ (\delta^{nlargest}_{dark,l}\ ) > \ \delta_{avg}\},$$

For a cell c with calcium traces δ:

If $|tw^{all}_{light,c}|$ > $|tw^{all}_{dark,c}|$, then cell c is light-responsive cell

else, cell c is dark-responsive cell.

If $\max(\delta^{all}_{light,l}) \in \delta^{qr}_{light,l}$ and $averag\ (\delta^{nlargest}_{light,l}\ ) > \ \delta_{avg}$, then cell c is transition from dark to light.

If $\max(\delta^{all}_{dark,l}) \in \delta^{qr}_{dark,l}$ and $averag\ (\delta^{nlargest}_{dark,l}\ ) > \ \delta_{avg}\}$, then cell c is transition from light to dark.

If $\delta^{1th}_{light,1} = \text{average}\left(\delta^{nlargest}_{light,1}\right)$, then cell c is active in first light phase.

If $\delta^{1th}_{dark,1} = \text{average}\left(\delta^{nlargest}_{dark,1}\right)$, then cell c is active in first dark phase.

Else, cell c does not classified any of above classes and assigned as unclassified.

---Classification of tail movement events

A customized script was used to extract four different kinds of tail swing events: struggle, left turn, right turn, and swim. In the developed algorithm, ϴ was denoted to the tail angle at time t and ϴ < 3 was set as baseline, whereas ϴ > 3 were used in the event classification process. The event length was determined by sliding time window W1 and W2. At first step swim (forward movement) events were identified by setting ϴ < 15 and W1 =50 (50 frames/500 milliseconds). The turn (flip) event was scored for unidirectional swing by setting ϴ > 15 and W2 = 200 (200 frames/1500 milliseconds), and further classified as right turn and left turn based on positive and negative tail angle values respectively. In the next step the struggle event was defined as vigorous bidirectional swings with ϴ > 15 and W2 = 200. The gap window ŧ = 100 was set and events within

the gap window were merged with hierarchy following swim-to-turn-to-struggle. The identified events were plotted on tail angle plots and cross checked for accuracy. The events were identified at 99% accuracy by manual curation with at least 3 subjects.

--- Relationship of CRF neuronal activity with tail movement events

CRF neurons that showed higher than average activity during at least 50% of the tail movement events were considered to have activity correlation with tail movement. Using this criterion, we found 101/117 CRF cells (from 7 larvae) showing activity correlated with tail movement. A simple algorithm to identify the peak (maximum) neuronal activity during the time window (+/-2 sec) around the onset of tail movement event was developed. The peak activity incidence was scored as concurrent, before or after the onset of tail movement (Fig. S5G). CRF neurons were classified as being active before, concurrent, or after tail movement if most peak activity incidences occur before, concurrent, or after the event (Fig. S5G').

---Analysis of altered photic tuning properties upon CRF^{Hy} neuronal ablation

Individual neurons were classified based on their photic tuning properties. The average percentage of cells corresponding to each photic response class in 147 anatomical mask regions (out of 294 from the Z-brain atlas) were compared between control and the CRF^{Hy} ablated groups (n=10 subjects for each group). The total number of cells recorded from each anatomical mask were also compared between control and the CRF^{Hy} ablated groups to verify equal data sampling. The anatomical areas showing differential photic representation (p <0.1) between control and the CRF^{Hy} ablated groups were identified. The statistical significance was further confirmed by non-parametric test and permutation test (10,000 repetitions) using a custom python script. The anatomical area was excluded if it was scored in less than 5 subjects either in control or the CRF^{Hy} ablated group. Anatomical areas that showed significant difference in total number of cells recorded were also excluded to avoid unintended bias in the comparison. The overall photic representation in an anatomical area showing significant differences between control and the CRF^{Hy} ablated groups were subsequently compared using the χ^2 test (p <0.1).

---Analysis of functional Connectivity

Functional connectivity comparison between control and CRFHy ablated groups was carried as described in Fig. 7A. The neuronal activity data (dF/F time series) for each subject from control and CRF^{Hy} ablated group was analyzed for functional connectivity. The cell-to-cell Pearson correlation matrix was derived at correlation thresholds ranging from 0.1 (low) to 0.8 (high). Since each cell has the anatomical mask identity, mask-to-mask correlation matrix was prepared. Large anatomical masks such as Telencephalon, Diencephalon were excluded. Based on our recorded data, 147 masks were analyzed, with correlation thresholds from 0.1 to 0.5. Increasing the threshold beyond 0.5 resulted in loss of anatomical masks from the analysis. For further comparison between control and CRF^{Hy}-ablated data, correlation matrix with 0.5 (moderate) threshold was therefore used. Anatomical regions that showed significant differences in total number of cells recorded between control and CRF^{Hy}-ablated groups were excluded. The mask-to-mask pair was excluded if it was

scored in less than 5 subjects either in control or CRF^{Hy}-ablated group. This resulted in a 135 x 135 mask-to-mask matrix. The correlation value for each mask pair was calculated (by averaging correlation value of all cell pairs within that mask pair). Out of 9,045 pairs, only 259 pairs were selected that showed significant difference in correlation value between control vs CRF^{Hy}-ablated groups (Unpaired t test $p<0.05$ and permutation test $p<0.05$). Similar to mask-to-mask correlation value, percent cells pairs with correlation above 0.5 in each mask pair was calculated and only those mask pairs were kept that showed significant difference between control vs CRF^{Hy}-ablated groups (Unpaired t test $p<0.05$ and permutation test $p<0.1$). Pairs showing increased and decreased functional connectivity were identified based on mean difference in correlation value and percent correlated cell pairs between control vs CRF^{Hy-}ablated groups.

References cited:

1. Bergeron SA, Carrier N, Li GH, Ahn S, Burgess HA. Gsx1 expression defines neurons required for prepulse inhibition. *Mol Psychiatry* 2015; **20:** 974-985.

2. Niell CM, Meyer MP, Smith SJ. In vivo imaging of synapse formation on a growing dendritic arbor. *Nat Neurosci* 2004; **7:** 254-260.

3. Vladimirov N, Mu Y, Kawashima T, Bennett DV, Yang C-T, Looger LL *et al.* Light-sheet functional imaging in fictively behaving zebrafish. *Nat Methods* 2014; **11**(9)**:** 883-884.

4. Lovett-Barron M, Chen R, Bradbury S, Andalman AS, Wagle M, Guo S *et al.* Multiple convergent hypothalamus-brainstem circuits drive defensive behavior. *Nat Neurosci* 2020; **23**(8)**:** 959-967.

5. Wee CL, Nikitchenko M, Wang WC, Luks-Morgan SJ, Song E, Gagnon JA *et al.* Zebrafish oxytocin neurons drive nocifensive behavior via brainstem premotor targets. *Nat Neurosci* 2019; **22:** 1477-1492.

6. Förster D, Kramer A, Baier H, Kubo F. Optogenetic precision toolkit to reveal form, function and connectivity of single neurons. *Methods* 2018; **150:** 42-48.

7. Fujimoto E, Stevenson TJ, Chien CB, Bonkowsky JL. Identification of a dopaminergic enhancer indicates complexity in vertebrate dopamine neuron phenotype specification. *Dev Biol* 2011; **352:** 393-404.

8. Kimura Y, Satou C, Fujioka S, Shoji W, Umeda K, Ishizuka T *et al.* Hindbrain V2a neurons in the excitation of spinal locomotor circuits during zebrafish swimming. *Curr Biol* 2013; **23:** 843-849.

9. Dal Maschio M, Donovan JC, Helmbrecht TO, Baier H. Linking Neurons to Network Function and Behavior by Two-Photon Holographic Optogenetics and Volumetric Imaging. *Neuron* 2017; **94:** 774-789.

10. Davison JM, Akitake CM, Goll MG, Rhee JM, Gosse N, Baier H *et al.* Transactivation from Gal4-VP16 transgenic insertions for tissue-specific cell labeling and ablation in zebrafish. *Dev Biol* 2007; **304:** 811-824.

11. Schindelin J, Arganda-Carreras I, Frise E, Kaynig V, Longair M, Pietzsch T *et al.* Fiji: an open-source platform for biological-image analysis. . *Nat Methods* 2012; **9:** 676-682.

12. Rohlfing T, Maurer CR. Nonrigid image registration in shared-memory multiprocessor environments with application to brains, breasts, and bees. *IEEE Trans Inform Technol Biomed* 2003; **7:** 16-25.

13. Giovannucci A, Friedrich J, Gunn P, Kalfon J, Brown BL, Koay SA *et al.* CaImAn an open source tool for scalable calcium imaging data analysis. *Elife* 2019; **8:** e38173.

14. van der Walt S, Colbert SC, Varoquaux G. The NumPy Array: A Structure for Efficient Numerical Computation. *Computing in Science & Engineering* 2011; **13:** 22-30.

15. Oliphant TE. Python for Scientific Computing. *Computing in Science & Engineering* 2007; **9:** 10-20.

16. van der Walt S, Schonberger JL, Nunez-Iglesias J, Boulogne F, Warner JD, Yager N *et al.* Scikit-image: image processing in Python. *Peer J* 2014; **2:** e453.

17. Pedregosa F, Varoquaux G, Gramfort A, Michel V, Thirion B, Grisel O *et al.* Scikit-learn: Machine Learning in Python. *JMLR* 2011; **12**(85)**:** 2825-2830.

18. Seabold S, Perktold J. statsmodels: Econometric and statistical modeling with python. *Proceedings of the 9th Python in Science Conference* 2010**:** 92-96.

19. Hunter JD. Matplotlib: A 2D Graphics Environment. *Computing in Science & Engineering* 2007; **9:** 90-95.

20. Kluyver T, Ragan-Kelley B, Pérez F, Granger B, Bussonnier M, Frederic J *et al.* Jupyter Notebooks – a publishing format for reproducible computational workflows. In: Loizides F, Scmidt B (eds). *Positioning and Power in Academic Publishing: Players, Agents and Agendas.* IOS Press2016, pp 87-90.

21. McKinney W, al e. Data structures for statistical computing in python. *Proceedings of the 9th Python in Science Conference* 2010; **445:** 51-56.

22. Kent WJ, Sugnet CW, Furey TS, Roskin KM, Pringle TH, Zahler AM *et al.* The Human Genome Browser at UCSC. *Genome Res* 2002; **12:** 996-1006.

23. Balciunas D, Wangensteen KJ, Wilber A, Bell J, Geurts A, Sivasubbu S *et al.* Harnessing a high cargo-capacity transposon for genetic applications in vertebrates. *PLoS Genet* 2006; **2:** e169.

24. Liu NA, Huang H, Yang Z, Herzog W, Hammerschmidt M, Lin S *et al.* Pituitary corticotroph ontogeny and regulation in transgenic zebrafish. *Mol Endocrinol* 2003; **17:** 959-966.

25. Bai Y, Liu H, Huang B, Wagle M, Guo S. Identification of environmental stressors and validation of light preference as a measure of anxiety in larval zebrafish. *BMC Neurosci* 2016; **17:** 63.

26. Wagle M, Mathur P, Guo S. Corticotropin-releasing factor critical for zebrafish camouflage behavior is regulated by light and sensitive to ethanol. *J Neurosci* 2011; **31:** 214-224.

27. Severi KE, Böhm UL, Wyart C. Investigation of hindbrain activity during active locomotion reveals inhibitory neurons involved in sensorimotor processing. . *Sci Rep* 2018; **8:** 13615.

Supplementary Figures and Tables

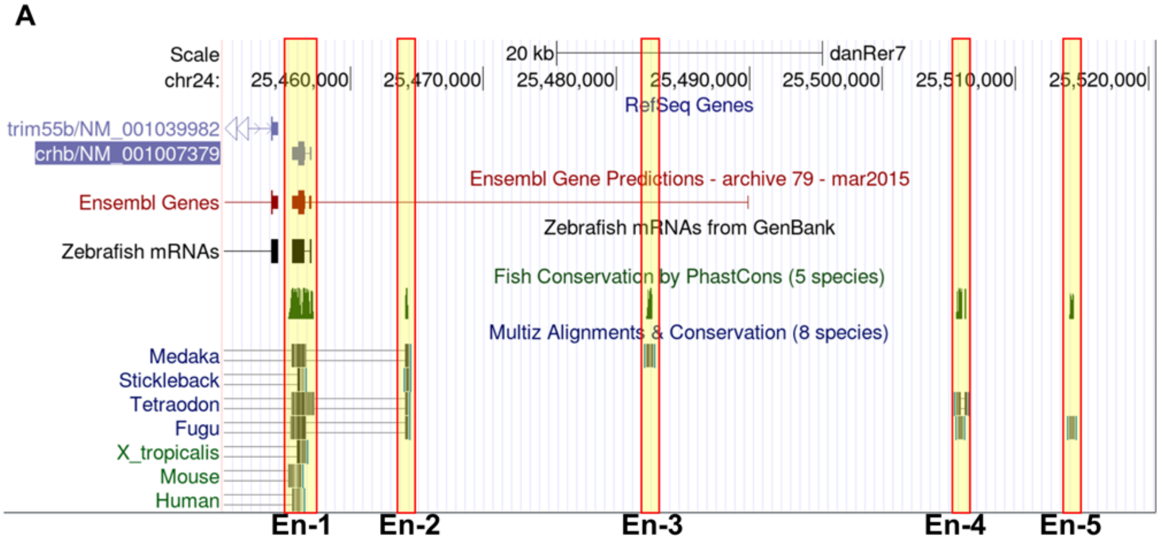

B

Conserved Element	Sense Primer Linker _Target	Antisense Primer Linker _Target	Amplicon size bp
En-1	**AATATAAA<u>GGATCC</u>**GAAAATACCCCTGGTTCTGTG	**ATGCTG<u>GCGGCCGC</u>**GTCAAAAAGTGGCGAGGAGAATC	725
En-2	**AATGTA<u>CCCGGG</u>**TATTCTTTATGAGTCTTATTAGC	**TAGAAC<u>GGATCC</u>**TGATTGATTGTAAAAGCG	351
En-3	**ATGGCA<u>CTCGAG</u>**ACAGTGTCAGACTATTTTACC	**AATGTA<u>CCCGGG</u>**TCTATATGAACAGGCGGC	478
En-4	**AGGCTA<u>CTGCAG</u>**GCTGTTTTCACAGTGAAGG	**ATGGCA<u>CTCGAG</u>**TTTAACAGGCACTATAGC	878
En-5	**GGACTG<u>AGATCT</u>**CTGCTGTGCAACCTAAATGC	**AGGCTA<u>CTGCAG</u>**TCTGGCATGAAACTATGG	425

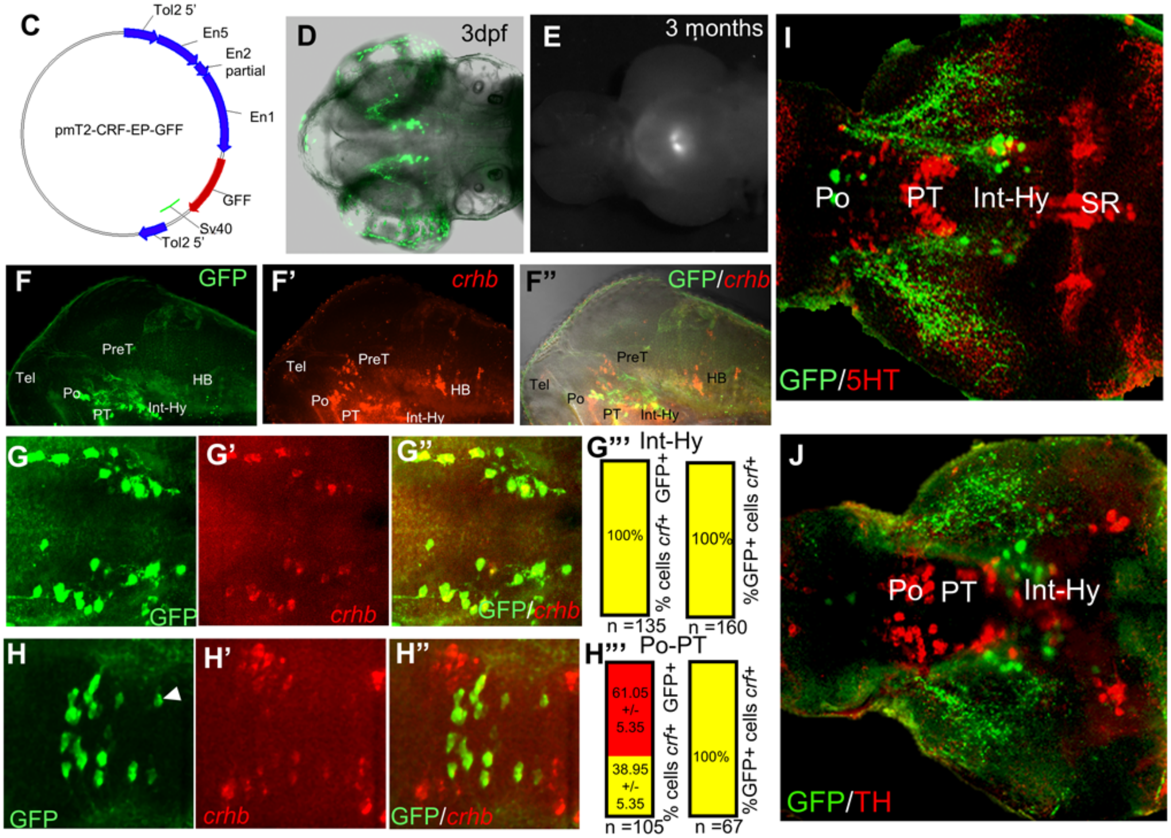

Fig. S1. An enhancer-based approach to gain specific genetic access to CRF^{Hy} neurons in zebrafish**. (A)** Image of the zebrafish *crhb* gene locus from the UCSC Genome browser, showing the position

of vertebrate conserved non-coding regions (yellow highlighted vertical boxes). **(B)** Table showing the primers used for amplifying conserved non-coding regions. **(C)** Schematic of the transgenic constructs. **(D)** GFP transgene expression in 3dpf larva. **(E)** GFP transgene expression in 3 months old adult brain –ventral view. **(F)** Lateral view Images of CRF^{Hy} neurons labeled with GFP (F) and *crhb* fluorescent in-situ hybridization (F’) and overlap with brightfield image (F”). **(G-H)** High magnification images prepared by maximum intensity projection of selected confocal z-planes showing dorsal view of CRF^{Hy} neurons in the intermediate hypothalamus (Int-Hy)(G-G’’) and pre-optic and posterior tuberculum (Po-PT) area (H-H’’) labeled with GFP (G&H), *crhb* fluorescent in-situ hybridization (G’&H’), and merged view (G’’&H’’). Quantification bar graphs (G’’’&H’’’) indicate endogenous *crhb* only-expressing cells (red) and cells expressing both endogenous *crhb* and transgene GFP (yellow). **(I-J)** Maximum intensity projection images of selected confocal z-planes showing dorsal view of 6dpf larval brain stained with anti-5HT (I) or anti-TH (J) (red stained cells) and anti-GFP antibody (green stained cells) showing relative location to serotonergic (I) and dopaminergic neurons (J) with respect to CRF^{Hy} neurons.

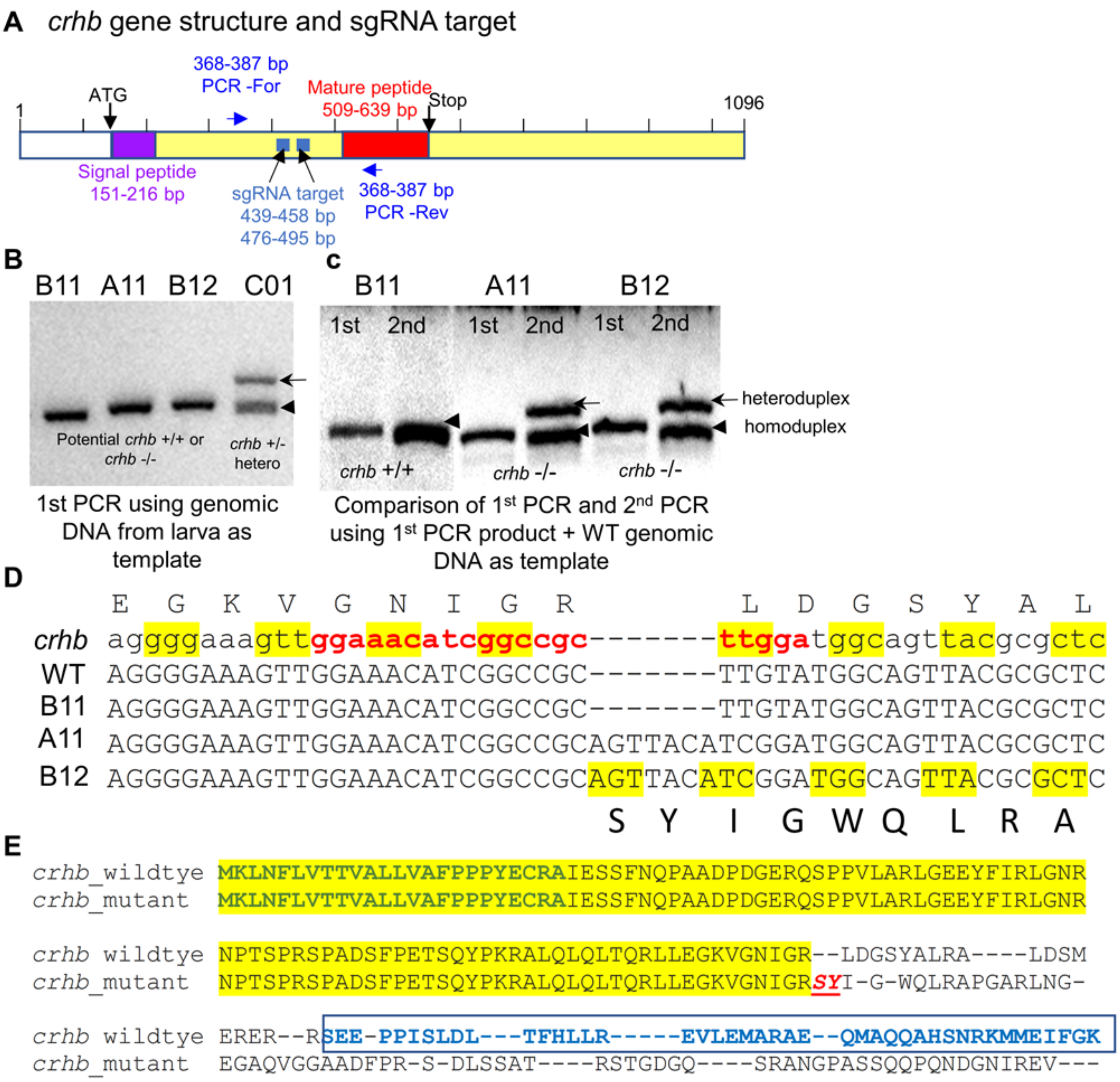

Fig. S2. Generation of the crhb* mutant *using CRISPR genome editing. **(A)** Schematic showing the *crhb* gene structure with the location of sgRNA target sites and genotyping primers indicated. **(B)** PCR amplification with primers flanking the mutation sites clearly identifies heterozygous (C01, with double bands). However, both wildtype and homozygous mutants have single bands, with minor mobility differences that are difficult to distinguish. **(C)** The 1st round PCR product from genotypically unknown individuals (B11, A11, B12) were mixed with PCR products from known wildtype and subjected to a single round of denaturation and annealing, and amplified again with primers flanking the mutation sites (2nd round PCR). The gel reveals the difference between wildtype and homozygous mutants: B11 widtype with single homoduplex band, versus A11 and B12 homozygous mutants showing double bands after the 2nd round of PCR. **(D)** Sequencing of the PCR product shows 7 bp insertion in the mutant, compared to the reference *crhb* sequence; the red letters show the guide

RNA target site. **(E)** Amino acid sequence comparison of wildtype and mutant *crhb,* with the sequences upstream of insertion site (marked with red letters) highlighted in yellow. The signal peptide is shown in green letters, sequence downstream of insertion site shows frame shift abolishing the production of mature CRF peptide (in blue bold letters).

A CMTK – registration of image stacks and assignment of ROI coordinates for Ca^{2+} imaging data

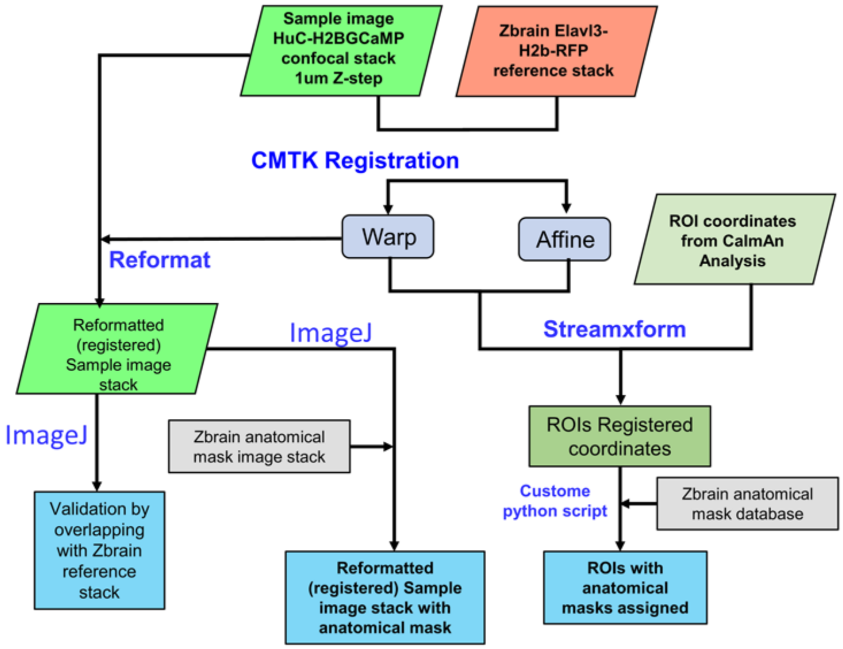

B Overlap of registered Tg[Huc-H2B-GCaMP] with Z-brain Elvl13-H2BRFP

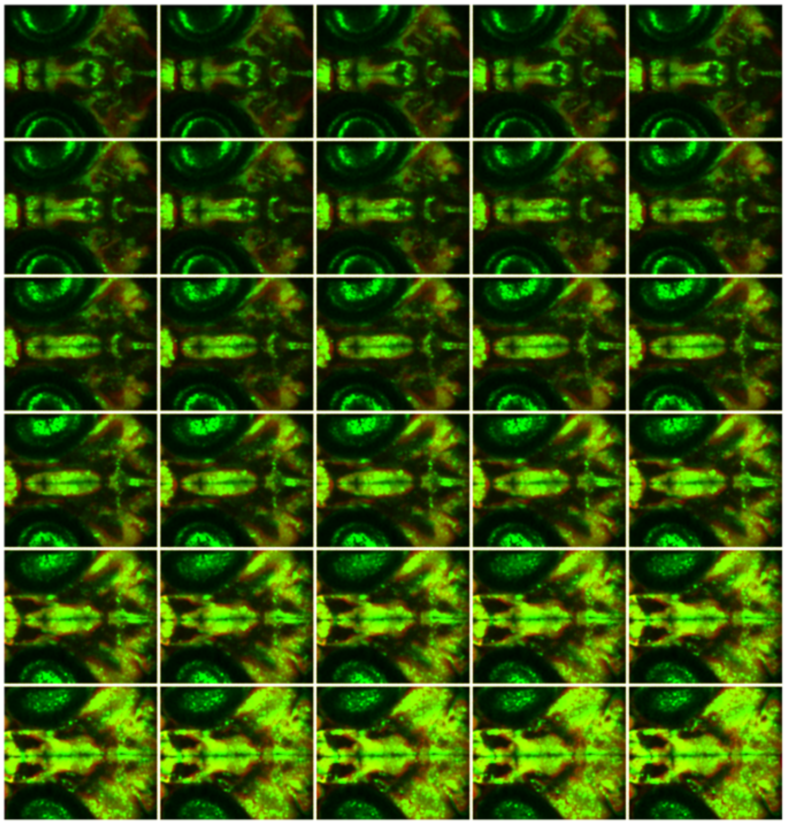

Fig. S3. Image registration with the Z-brain atlas. **(A)** A flow chart showing registration of image stacks using the CMTK-reformat function to identify anatomical areas based on the Z-brain atlas anatomical masks. The CMTK-streamxform is used for registering coordinates of ROIs from CaImAn analysis and custom script

is designed to assign Z-brain anatomical masks. **(B)** Montage of z-slices from composite image stacks created by overlapping Elval3-H2B-RFP image stacks from Z-brain (red) and HuC::H2b-GCaMP stacks (green) after CMTK registration. Excellent alignments are obtained.

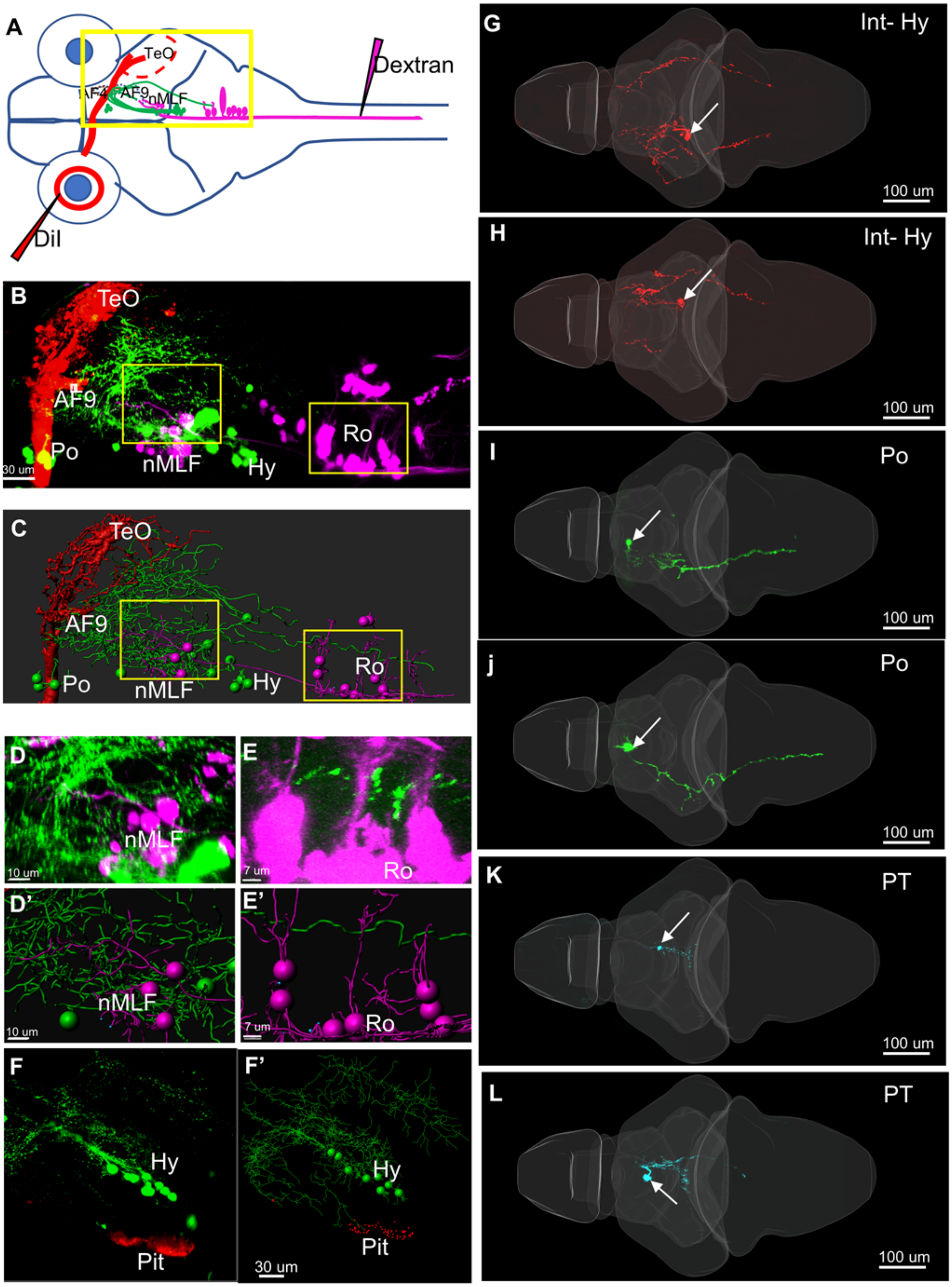

Fig. S4. Tracing single CRF^{Hy} neuronal projections. **(A)** Schematic showing the procedure of DiI labeling of retinal ganglion cells (RGCs) in the eye and back-filling spinal cord in transgenic animals. **(B)** Image of retinal DiI (red), CRF-GFP (green), along with nMLF and reticulospinal neurons (magenta) backfilled with

Alexa-Dextran in the spinal cord. **(C)** Imaris rendering of the image in (b). **(D-D’, E-E’)** zoomed-in image and Imaris rendering showing CRF-GFP labeled processes in proximity to nMLF (D,D’) and reticulospinal neurons (e,e’). **(F-F’)** Maximum intensity projection of confocal stacks and Imaris rendering of immunostaining of CRF^{Hy} transgenic larvae with anti-GFP antibody (green) and anti-ACTH (red), showing CRF^{Hy} neuronal processes in close proximity to pituitary cells labelled with anti-ACTH antibody. **(G-L)** Examples of single CRF neuron labeling, arrow pointing to the cell soma. CRF neurons in Int-Hy (G,H), Pre-optic area (I,J) and Posterior Tuberculum (K,L).

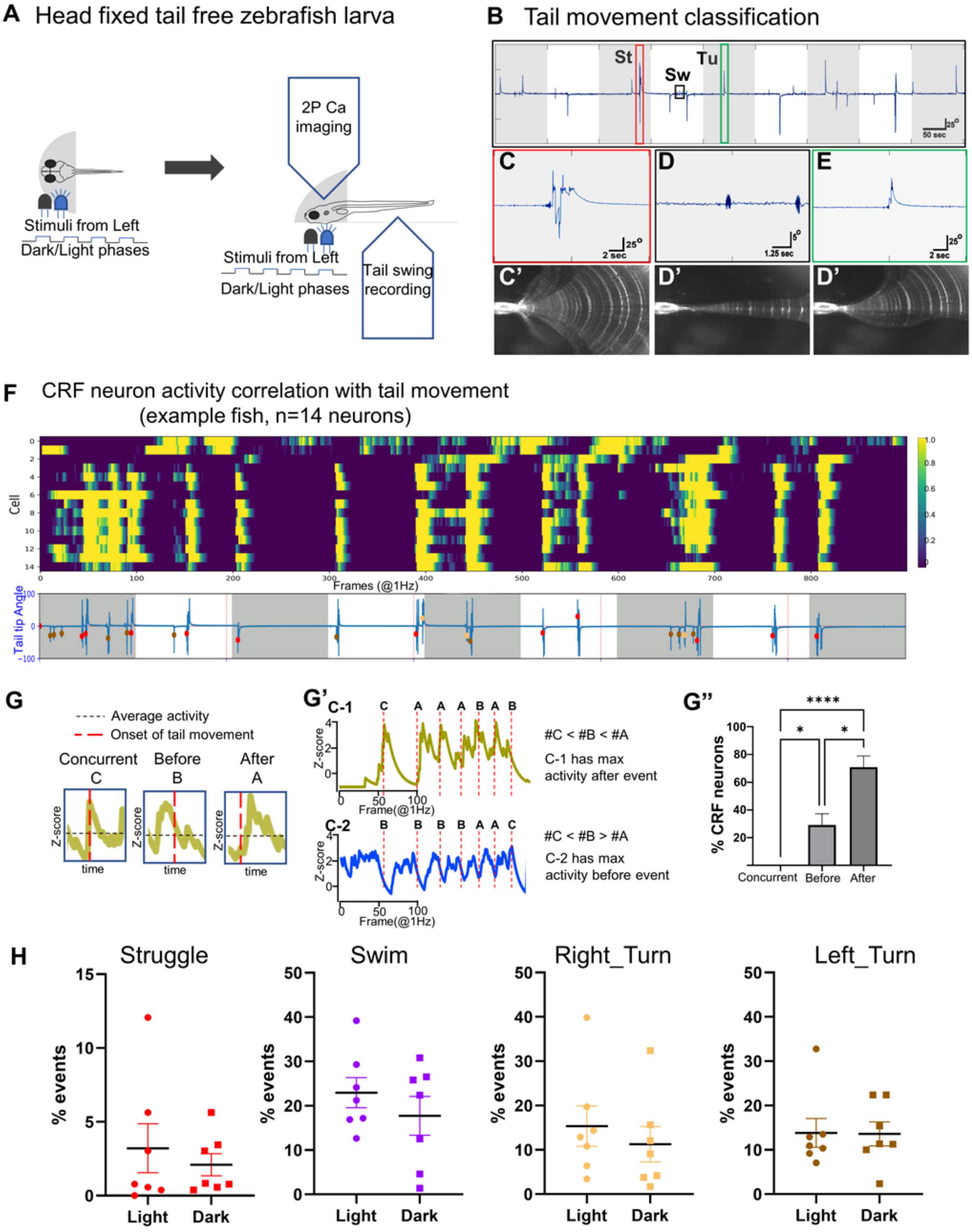

Fig. S5. CRF^{Hy} neurons in head-restrained/tail-free non-paralyzed larval zebrafish are strongly activated during vigorous motion. **(A)** Schematic showing calcium imaging in non-paralyzed and tail-free larva. The blue LED (455 nm) light was pulsed to deliver photic stimuli in alternate light and dark phases. Sub-

stage objective lens and camera was used to record tail movements. **(B)** Example tail tip deflection angle (y-axis) plot, showing three types of events St-Struggle, Sw-Swim, Tu-Turn (x-axis: time). **(C-E)** Enlarged view of plot insets in B; **(C'-E')** maximum intensity projection of selected video frames during Struggle, Swim, and Turn events. **(F)** An example Z-score heatmap plot of CRF^{Hy} neuronal activity imaged in non-paralyzed, tail free larva showing correlation with tail movement events shown in the tail tip angle plot below. **(G-G")** Correlation of CRF neuronal activity with tail movement. Schematic showing peak neuronal activity in relation to the onset of tail movements (G). Schematic showing individual neuron classification based on the numbers of concurrent, before and after events (G'). Neuron c-1 is classified as "after" since most events show CRF neuronal activity after tail movements, whereas c-2 is classified as "before". Concurrent activity events were found to be rare hence none of the CRF neurons were classified as "concurrent". Plot on right shows percent of CRF neurons with activity in relation to the tail movements (G"), Kruskal-Wallis test, Dunn's multiple comparisons test, n= 101 neurons, * p<0.05, **** p<0.0001, error bars representing SEM. **(H)** Scatter plots showing average number of struggles, swim, right or left turns in light and dark phases, n=7 larvae, error bars representing SEM. No significant difference was observed for any of four events.

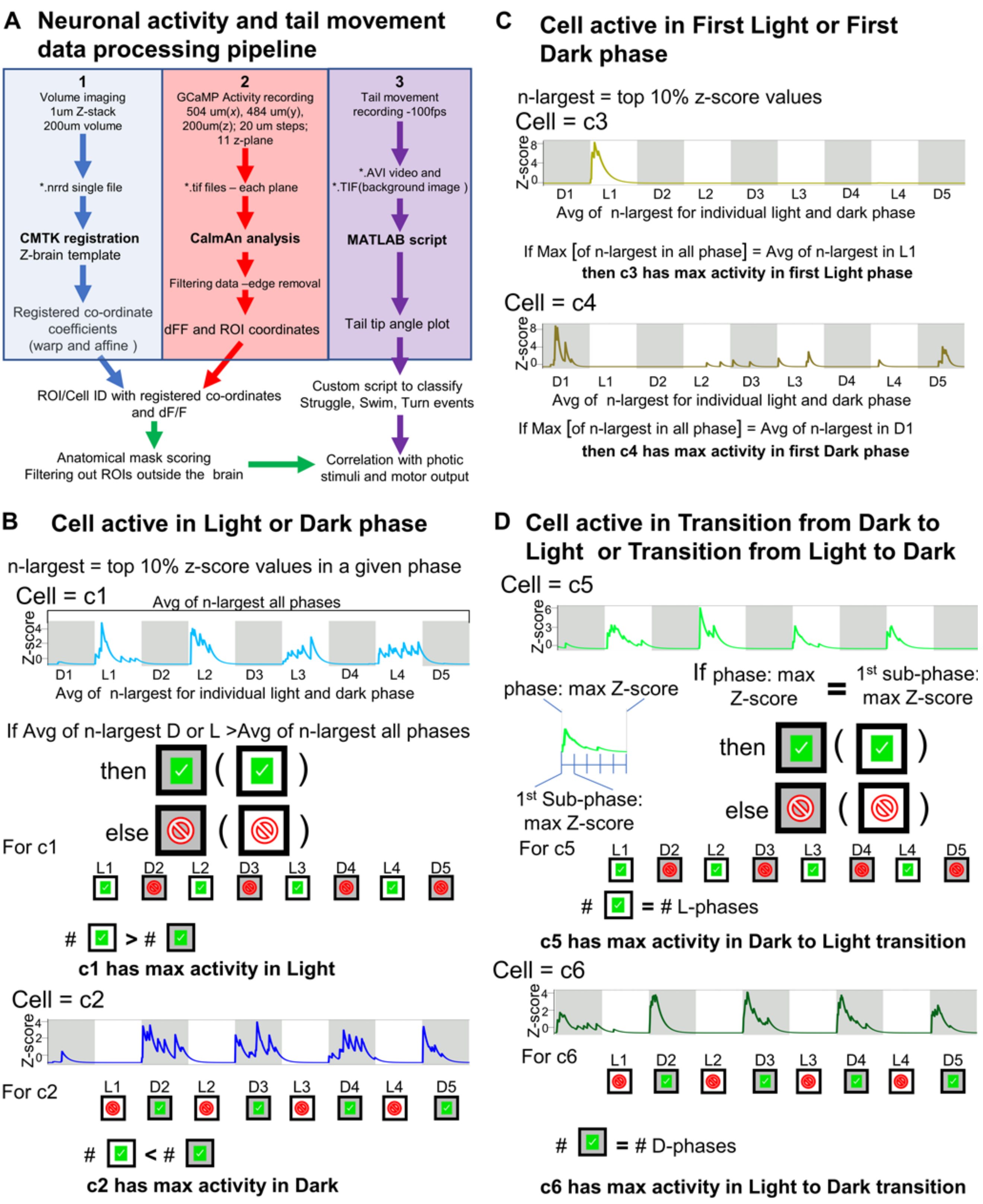

Fig. S6. Brain-wide data processing pipeline and the algorithm for classifying photic response neuronal types. **(A)** Flow chart showing three-way data processing for each larva. The HuC-H2B-CaMP6s image stacks with 1um z-step was registered with the Z-brain Elvl13-H2B-RFP template through CMTK

registration (1). The calcium imaging data were analyzed using the CaImAn software (2) and tail movement recording was processed using Matlab and custom python scripts (3). **(B-D)** Classification of neurons based on their photic responses. Maximum activity of neurons with respect to light and dark phases was scored. **(B)** The n-largest are the top 10% z-score values of each neuronal activity plot in a given phase. The example activity plot (z-score) with light and dark phase is shown at the top. The average n-largest of each light phase (L1-L3) and dark phase (D2-D4) were compared with average n-largest of entire data (in all phases). The example cell c1 is classified to "active in light" class, as the number of light phases showing avg-n-largest higher than avg-n-larges-all are more than the number of dark phases with avg-n-largest higher than avg-n-larges-all. The example cell c2 is classified to "active in dark" class, as number of dark phases showing avg-n-largest higher than avg-n-larges-all are more than light phases with avg-n-largest higher than avg-n-larges-all. **(C)** Neurons were classified in "First Light" (example cell c3) or "First Dark" (example cell c4) by comparing maximum values of n-largest-all with avg-n-largest in first light (L1) or first dark (D1) phase. **(D)** Each light and dark phase is divided into 5 sub-phases, if the maximum z-score value of the phase happens to be in the first sub-phase then that phase is considered as active during transition. The neuron is classified to "Transition from Dark to Light" class if number of light phases are more than number dark phases active during transition as shown in example cell c5. The example cell c6 is classified to "Transition from Light to Dark" class as number of dark phases active during transition are more than light phases.

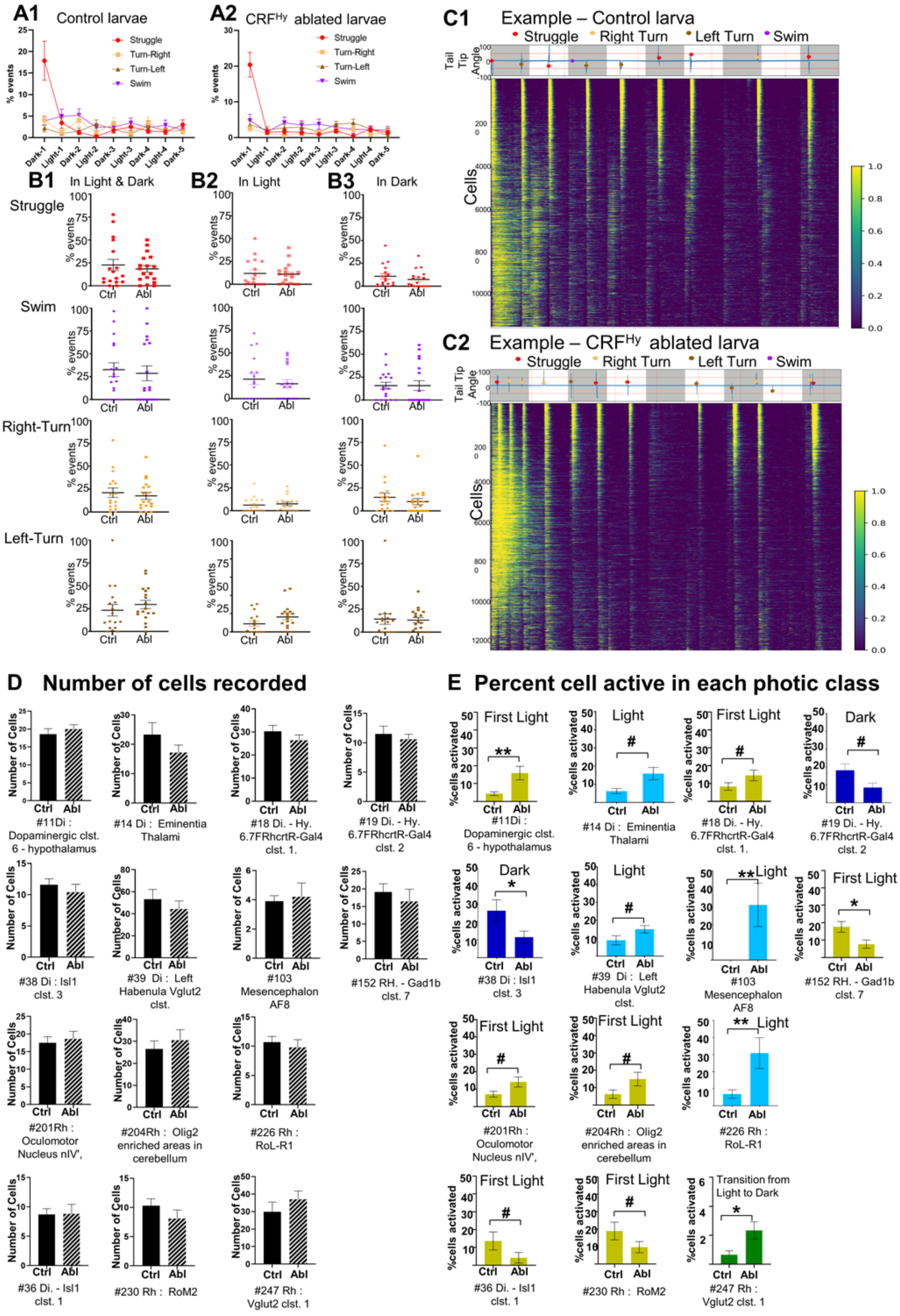

***Fig. S7*. Comparison of tail movement events and brain-wide neuronal activity between control and CRF^Hy-ablated subjects. (A1, A2)** Tail tip turn events in all light and dark phases for control (A1) and CRF^Hy-ablated subjects (A2). **(B1-B3)** Comparison of struggle, swim, right turn and left turn events between control

and CRF^{Hy}-ablated subjects during all phases except the first dark phase (B1), all light phases (B2), and all but first dark phases (B3). No significant differences were observed. Mann Whitney test, n=17 control, 18 CRF^{Hy}-ablated, error bars representing SEM. **(C1,C2)** Example of brain wide activity heatmaps arranged in order of correlation with tail moment for control (C1) and CRF^{Hy}-ablated larva (C2). The top shows the tail movement plot with tail tip angle and light/dark phases marked. Color dots indicate the type of tail moment events. **(D)** comparison of total number of cells recorded between control and CRF^{Hy}-ablated subjects for anatomical regions from Fig. 6B, E. No significant differences were observed. n=10 larvae per group, non-parametric two tailed Mann Whitney test **(E)** Comparison cells activated in a photic class between control and CRF^{Hy}-ablated subjects. The color of the bar graph is as per photic class indicated in Fig. 6B. n=10 larvae per group, non-parametric two tailed Mann Whitney test, # p<0.1, *p<0.05, ** p<0.01, error bars representing SEM.

Table S1. Increased functional connectivity in CRF^{Hy} -ablated subjects

Mask-A			Mask-B			Correlation value (R)				% cell pairs with R above threshold (0.5)			
Name	Neuro-transmitter	Z-brain Mask ID	Name	Neuro-transmitter	Z-brain Mask ID	Ctrl	CRF^{Hy} Abl	Unpaired t-test	permutation one sided	Ctrl-% cell pair	CRF^{Hy} Abl-% cell pair	Unpaired t test	permutation one sided
Mes. - Ptf1a clst.	GABA	101	Di. - Ant. pretectum clst. of vmat2 Neurons	Glut	4	0.603	0.621	*	*	9.606	14.714	*	*
Mes. - Ptf1a clst.	GABA	101	Di. - Dopaminergic clst. 3 - hypothalamus	-	9	0.578	0.622	*	**	6.351	10.610	*	**
Mes. - Ptf1a clst	GABA	101	Rh. - Cerebelluar-VGlut2 enriched areas	-	130	0.614	0.630	*	*	7.653	10.410	*	**
Mes. - Ptf1a clst.	GABA	101	Rh. - Cerebellum	-	131	0.601	0.618	**	*	5.292	8.749	**	**
Mes. - Ptf1a clst.	GABA	101	Rh. - Eminentia Granularis	GABA	134	0.600	0.625	*	*	6.215	10.384	*	**
Mes. - Ptf1a clst.	GABA	101	Rh. - Gad1b clst. 2	GABA	146	0.606	0.629	**	**	8.634	14.933	**	**
Mes. - Ptf1a clst.	GABA	101	Di. - Hy. VGlut2 clst. 5	Glut	31	0.588	0.619	*	*	6.727	13.027	*	*
Rh. - Otpb clst 2 - LC	NA -	206	Di. - Hb.	Glut	15	0.581	0.608	*	*	2.003	4.621	*	*
Rh. - Otpb clst 2 -LC	-	206	Di. - Right Hb. VGlut2 clst.	Glut	73	0.579	0.616	*	*	2.189	6.467	*	*
Rh. - Otpb clst2 -LC	-	206	Rh. - Otpb clst. 1	-	205	0.607	0.681	**	*	11.045	32.439	**	#
Di. - Right Hb. VGlut2 clst.	Glut	73	Rh. - RoL-R1	-	226	0.580	0.627	*	*	2.326	6.470	*	*
Rh. - Otpb clst. 1	-	205	Di. - Hy. - Ca. Hy. Neural clst.	-	16	0.576	0.615	*	*	2.844	7.756	*	#
Di. - Hy. Qrfp neuron clst	Glut	26	Rh. - Gad1b clst. 16	GABA	142	0.572	0.591	*	*	2.221	5.549	*	*
Rh. - Gad1b clst. 1	GABA	135	Rh. - Gad1b clst. 7	GABA	152	0.575	0.600	**	*	2.616	6.249	**	*

# p<0.1, * p<0.05, **p<0.01

Table S2. Decreased functional connectivity in CRF^{Hy} -ablated subjects

Mask-A			Mask-B			Correlation value (R)				% cell pairs with R above threshold (0.5)			
Name	Neuro-transmitter	Z-brain Mask ID	Name	Neuro-transmitter	Z-brain Mask ID	Ctrl	CRF^{Hy} Abl	Unpaired t-test	permutation one sided	Ctrl-% cell pair	CRF^{Hy} Abl - % cell pair	Unpaired t test	permutation one sided
Mes. - Vmat2 clst. of paraventricular organ	-	112	Di. - Otpb clst. 4	-	49	0.626	0.594	*	*	7.866	4.217	*	*
Mes. - Vmat2 clst. of paraventricular organ	-	112	Mes. - Retinal Arborization Field 9 (AF9)	-	104	0.644	0.575	*	**	5.610	2.603	*	*
Mes. - Vmat2 clst. of paraventricular organ	-	112	Rh. - Ant. clst. of nV Trigeminal Motorneurons	-	124	0.621	0.594	*	*	11.543	5.287	*	**
Mes. - Vmat2 clst. of paraventricular organ	-	112	Rh. - Cerebelluar-VGlut2 enriched areas	Glut	130	0.637	0.604	*	*	7.722	4.062	*	*
Di. - Dopaminergic clst. 6 - hypothalamus	Glut	11	Di. - Pretectal Gad1b clst.	-	64	0.594	0.572	*	*	4.737	2.435	*	*
Di. - Dopaminergic clst. 6 - hypothalamus	Glut	11	Mes. - Otpb clst.	-	99	0.605	0.568	*	**	4.641	2.513	*	*
Mes. - Medial Tectal Band	-	96	Di. - Pretectal DA. clst.	-	65	0.625	0.615	*	*	14.482	9.642	*	*
Mes. - Medial Tectal Band	-	96	Di. - Olig2 Band	Glut	43	0.629	0.613	*	*	12.083	7.955	*	*
Di. - Hy. 6.7FRhcrtR-Gal4 clst. 1	Glut	18	Rh. - Cerebellum	-	131	0.606	0.586	*	**	4.120	2.777	*	*
Di. - Hy. 6.7FRhcrtR-Gal4 clst. 2	Glut	19	Rh. - Interpeduncular Nucleus	-	176	0.608	0.571	*	**	4.100	1.448	*	**
Di. - Isl1 clst. 1	Glut	36	Di. - Retinal Arborization Field 6 (AF6)	-	72	0.609	0.569	*	*	9.230	4.623	*	#
Di. - Migrated Area of the PreTeO. (M1)	-	41	Tel. - Isl1 clst. 1	-	277	0.590	0.555	*	**	2.205	0.660	*	*

# $p<0.1$, * $p<0.05$, ** $p<0.01$

469 **Table S3. Anatomical regions – Z-brain mask ID, short labels and abbreviations**

Z-brain mask ID	Anatomical labels	Short label (Fig 6, Fig 7, Fig S7, Table S1 & S2	Abbreviation (Fig1, Fig 3)
1	u'Diencephalon -',		Di
4	u'Diencephalon - Anterior pretectum cluster of vmat2 Neurons',	Di. - Ant. pretectum clst. of vmat2 Neurons	
5	u'Diencephalon - Caudal Hypothalamus',		Ca-Hy
8	u'Diencephalon - Dopaminergic Cluster 2 - posterior tuberculum',		DA clust-2
9	u'Diencephalon - Dopaminergic Cluster 3 - hypothalamus',	Di. - Dopaminergic clst. 3 - hypothalamus	
11	u'Diencephalon - Dopaminergic Cluster 6 - hypothalamus',	Di. - Dopaminergic clst. 6 - hypothalamus	
14	u'Diencephalon - Eminentia Thalami',	Di - Eminentia Thalami	EmT
15	u'Diencephalon - Habenula',	Di. - Hb.	Hb
16	u'Diencephalon - Hypothalamus - Caudal Hypothalamus Neural Cluster',	Di. - Hy. - Ca. Hy. Neural clst.	
17	u'Diencephalon - Hypothalamus - Intermediate Hypothalamus Neural Cluster',		Int-Hy-Neural-clust
18	u'Diencephalon - Hypothalamus 6.7FRhcrtR-Gal4 cluster 1',	Di. - Hy. 6.7FRhcrtR-Gal4 clst. 1	
19	u'Diencephalon - Hypothalamus 6.7FRhcrtR-Gal4 cluster 2',	Di. - Hy. 6.7FRhcrtR-Gal4 clst. 2	
26	u'Diencephalon - Hypothalamus Qrfp neuron cluster',	Di. - Hy. Qrfp neuron clst	
31	u'Diencephalon - Hypothalamus Vglut2 Cluster 5',	Di. - Hy. VGlut2 clst. 5	
35	u'Diencephalon - Intermediate Hypothalamus',		Int-Hy
36	u'Diencephalon - Isl1 cluster 1',	Di. - Isl1 clst. 1	
38	u'Diencephalon - Isl1 cluster 3',	Di - Isl1 cluster 3	
39	u'Diencephalon - Left Habenula Vglut2 Cluster',	Di - Left Habenula Vglut2 Cluster	
46	u'Diencephalon - Otpb Cluster 1',		Otpb-clust1
49	u'Diencephalon - Otpb Cluster 4',		Otpb-clust4
50	u'Diencephalon - Oxtl Cluster 1 in Preoptic Area',		Oxtl-clust1
53	u'Diencephalon - Oxtl Cluster 4 - sparse in hypothalamus',		Oxtl-clust4
57	u'Diencephalon - Pituitary',		Pit
58	u'Diencephalon - Posterior Tuberculum',		PT
60	u'Diencephalon - Preoptic Area',		Po
66	u'Diencephalon - Pretectum',		PreT
70	u'Diencephalon - Retinal Arborization Field 4 (AF4)',		AF4
72	u'Diencephalon - Retinal Arborization Field 6 (AF6)',		AF6
94	u'Mesencephalon -',		Mes

97	u'Mesencephalon - NucMLF (nucleus of the medial longitudinal fascicle)',		nMLF
102	u'Mesencephalon - Retinal Arborization Field 7 (AF7)',		AF7
103	u'Mesencephalon - Retinal Arborization Field 8 (AF8)',	Mes - Retinal Arborization Field 8 (AF8)	AF8
104	u'Mesencephalon - Retinal Arborization Field 9 (AF9)',	Mes. - Retinal Arborization Field 9 (AF9)	AF9
110	u'Mesencephalon - Torus Semicircularis',		Ts
112	u'Mesencephalon - Vmat2 cluster of paraventricular organ',	Mes. - Vmat2 clst. of paraventricular organ	
114	u'Rhombencephalon -',		Rh
124	u'Rhombencephalon - Anterior Cluster of nV Trigeminal Motorneurons',	Rh. - Ant. clst. of nV Trigeminal Motorneurons	
130	u'Rhombencephalon - Cerebelluar-Vglut2 enriched areas',	Rh. - Cerebelluar-VGlut2 enriched areas	
131	u'Rhombencephalon - Cerebellum',	Rh. - Cerebellum	
134	u'Rhombencephalon - Eminentia Granularis',	Rh. - Eminentia Granularis	
135	u'Rhombencephalon - Gad1b Cluster 1',	Rh. - Gad1b clst. 1	
142	u'Rhombencephalon - Gad1b Cluster 16',	Rh. - Gad1b clst. 16	
146	u'Rhombencephalon - Gad1b Cluster 2',	Rh. - Gad1b clst. 2	
152	u'Rhombencephalon - Gad1b Cluster 7',	Rh. - Gad1b clst. 7	
176	u'Rhombencephalon - Interpeduncular Nucleus',	Rh. - Interpeduncular Nucleus	IPN
201	u'Rhombencephalon - Oculomotor Nucleus nIV',	Rh - Oculomotor Nucleus nIV	
204	u'Rhombencephalon - Olig2 enriched areas in cerebellum',	Rh - Olig2 enriched areas in cerebellum	
205	u'Rhombencephalon - Otpb Cluster 1',	Rh. - Otpb clst. 1	
206	u'Rhombencephalon - Otpb Cluster 2 - locus coeruleus',	Rh. - Otpb clst. 2 - locus coeruleus	
218	u'Rhombencephalon - Raphe - Superior',		SR
226	u'Rhombencephalon - RoL-R1',	Rh. - RoL-R1	RoL-R1
227	u'Rhombencephalon - RoL2',		RoL2
228	u'Rhombencephalon - RoL3',		RoL3
229	u'Rhombencephalon - RoM1',		RoM1
230	u'Rhombencephalon - RoM2',	Rh - RoM2	
247	u'Rhombencephalon - Vglut2 cluster 1',	Rh - Vglut2 cluster 1	
275	u'Telencephalon -',		Tel
277	u'Telencephalon - Isl1 cluster 1',	Tel. - Isl1 clst. 1	
	Hindbrain		HB
	Reticulospinal neurons		Ro

Video S1. A video clip shows a free-swimming larval zebrafish in the light dark choice chamber. Recorded with infrared camera; the light zone, dark zone, and the virtual border zone are marked with light blue, dark blue, and orange rectangles respectively.

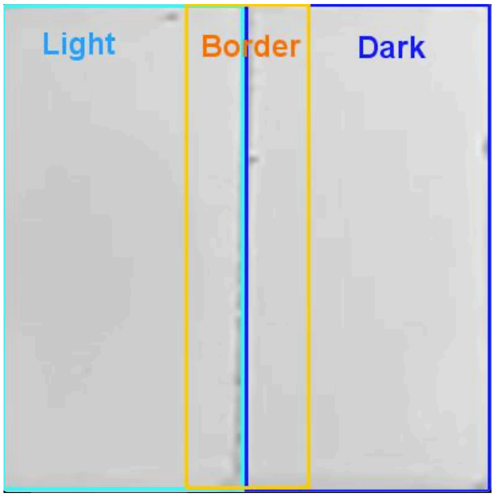

Video S2. A video clip shows the Imaris rendering of single hypothalamic CRF neuron project patterns. Three neurons, marked red (in Int-Hy), green (in PT), and blue (in PO) are shown.

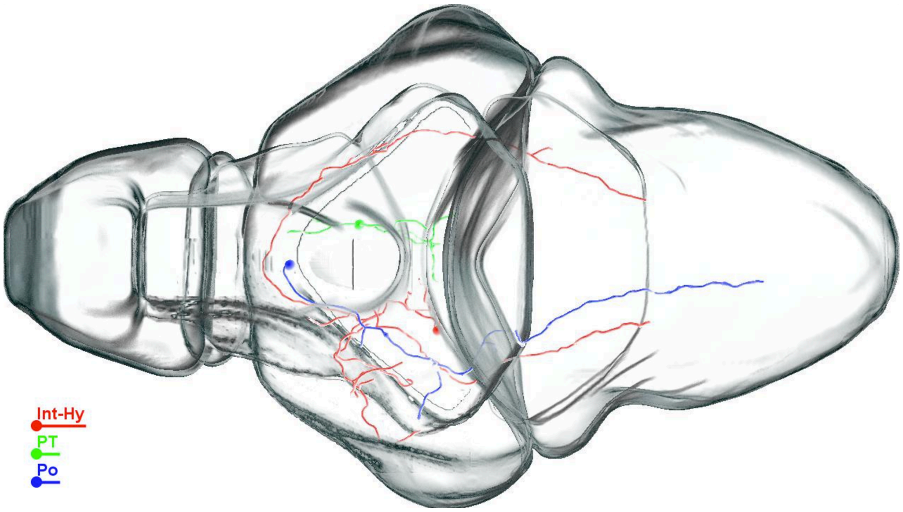

Video S3. A video clip shows *in vivo* calcium imaging of a single Z-plane in a 6-7 dpf larval zebrafish brain during ~15-min. Dark period, light period, and tail movements are marked.

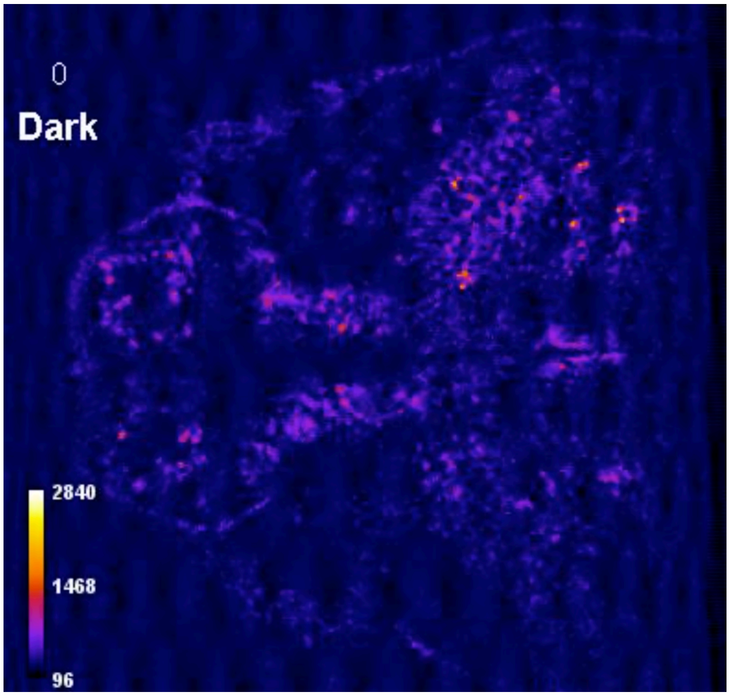

Video S4. A video clip shows the brain areas with increased (red) or decreased (blue) function connectivity in CRF^{Hy} neuron-ablated subjects compared to control siblings.

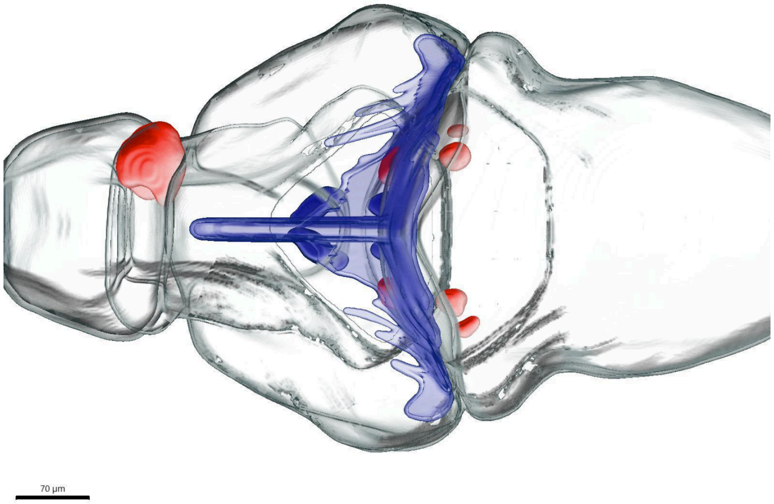

Supplement: Supplementary file 5 — Supplemental material [file 41380_2022_1567_MOESM5_ESM.pdf]
